# Supplementary material for: Regulation of Drosophila Brain Wiring by Neuropil Interactions via a Slit-Robo-RPTP Signaling Complex
Source: Dev Cell. 2016 Oct 24;39(2):267–78. doi: 10.1016/j.devcel.2016.09.028 (PMC5084709; doi:10.1016/j.devcel.2016.09.028)
Supplement: Document S1. Supplemental Experimental Procedures and Figures S1–S6 [file mmc1.pdf]

Developmental Cell, Volume *39*

Supplemental Information

Regulation of *Drosophila* Brain Wiring by Neuropil Interactions via a Slit-Robo-RPTP Signaling Complex

Carlos Oliva, Alessia Soldano, Natalia Mora, Natalie De Geest, Annelies Claeys, Maria-Luise Erfurth, Jimena Sierralta, Ariane Ramaekers, Dan Dascenco, Radoslaw K. Ejsmont, Dietmar Schmucker, Natalia Sanchez-Soriano, and Bassem A. Hassan

Supplemental Inventory

Supplemental Figure S1, Related to Figure 1.

Supplemental Figure S2, Related to Figure 2.

Supplemental Figure S3, Related to Figure 3.

Supplemental Figure S4, Related to Figure 4.

Supplemental Figure S5, Related to Figure 6.

Supplemental Figure S6, Related to Figure 7.

Supplemental Figure legends

Supplemental Experimental Procedures

Supplemental References

Figure S1, related to Figure 1

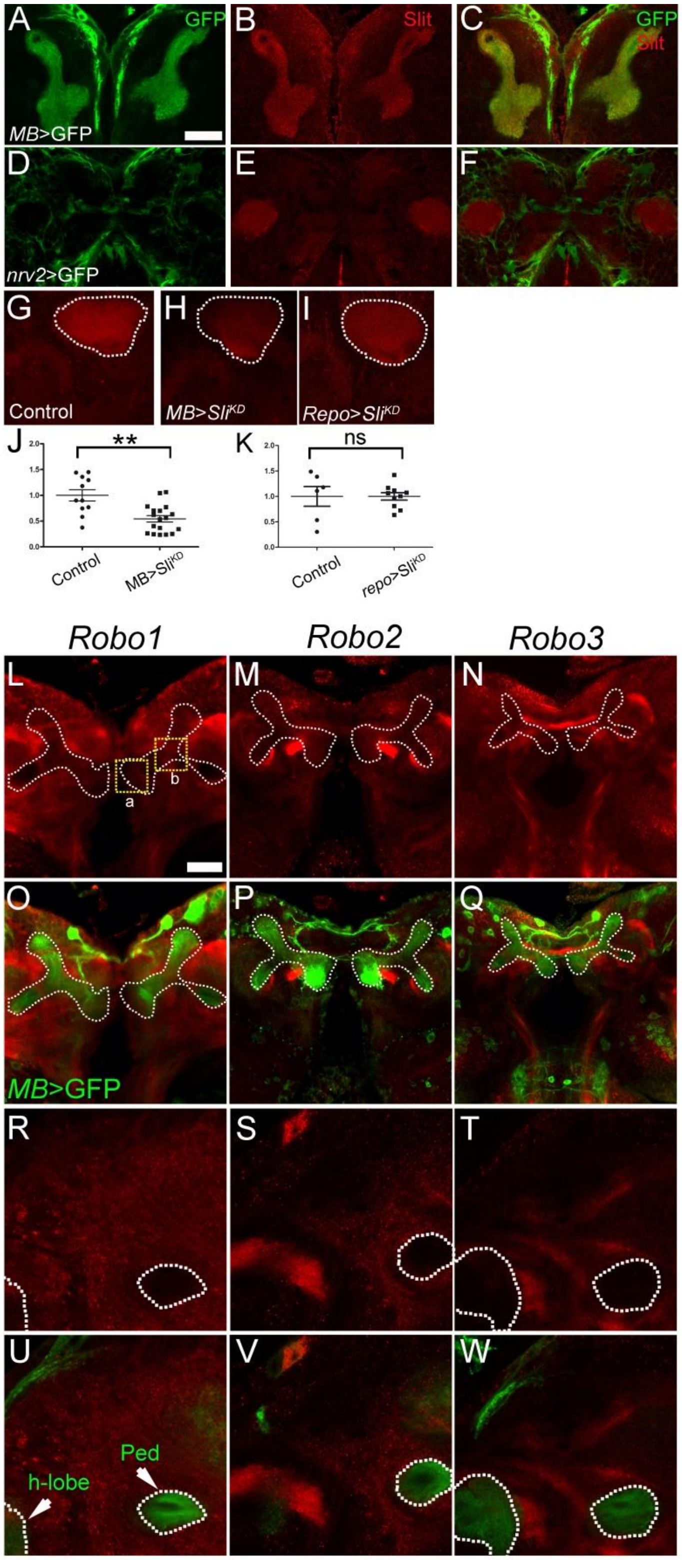

Figure S2, related to Figure 2

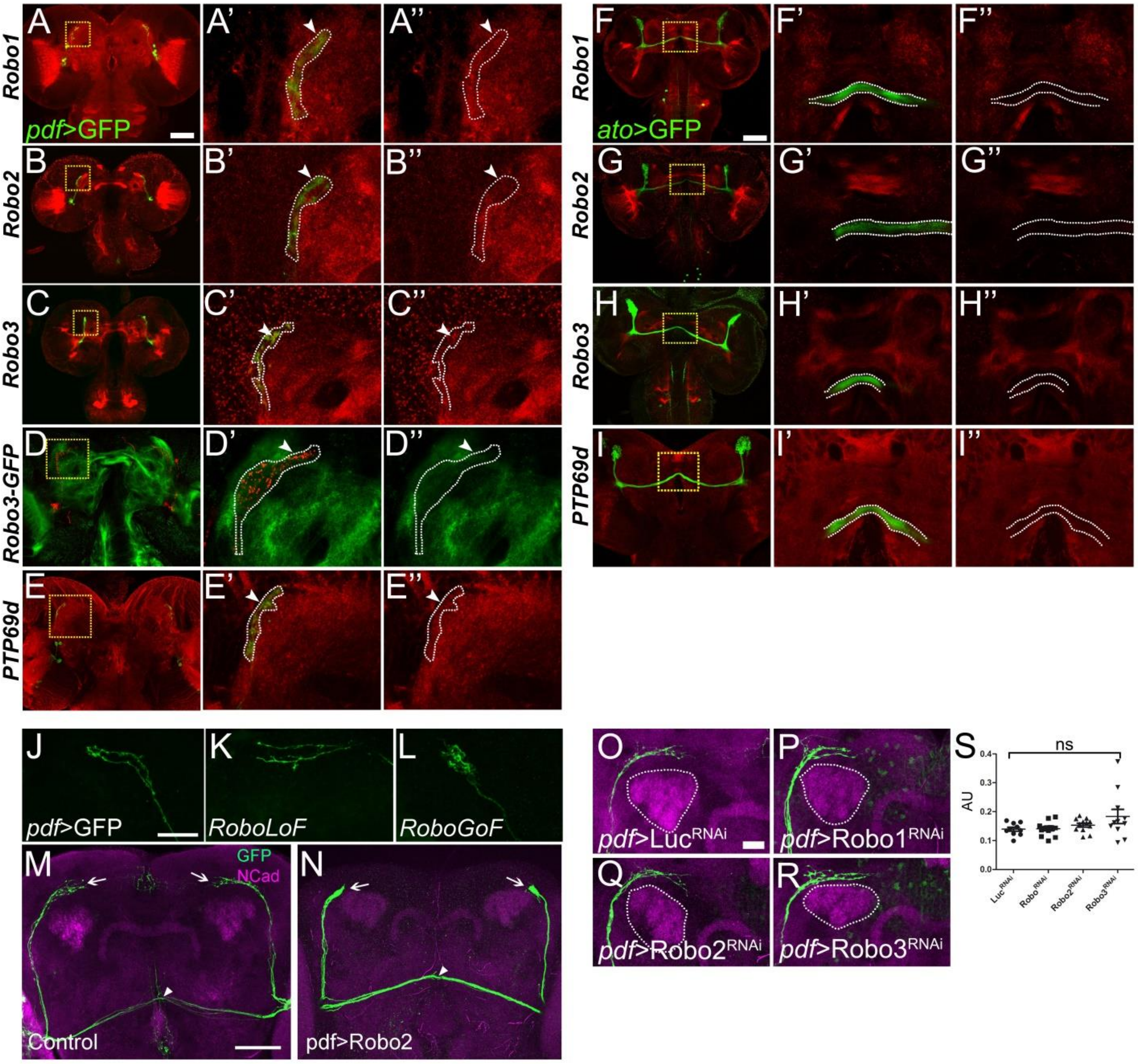

Figure S3, related to Figure 3

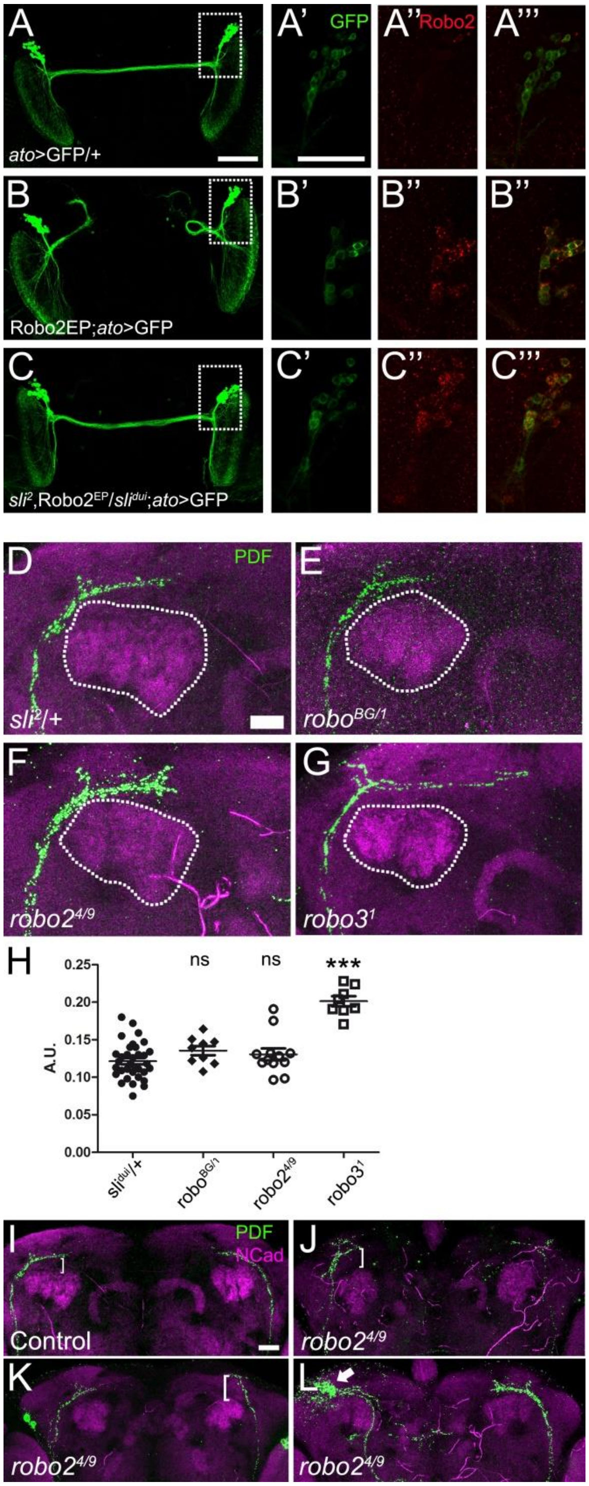

Figure S4, related to Figure 4

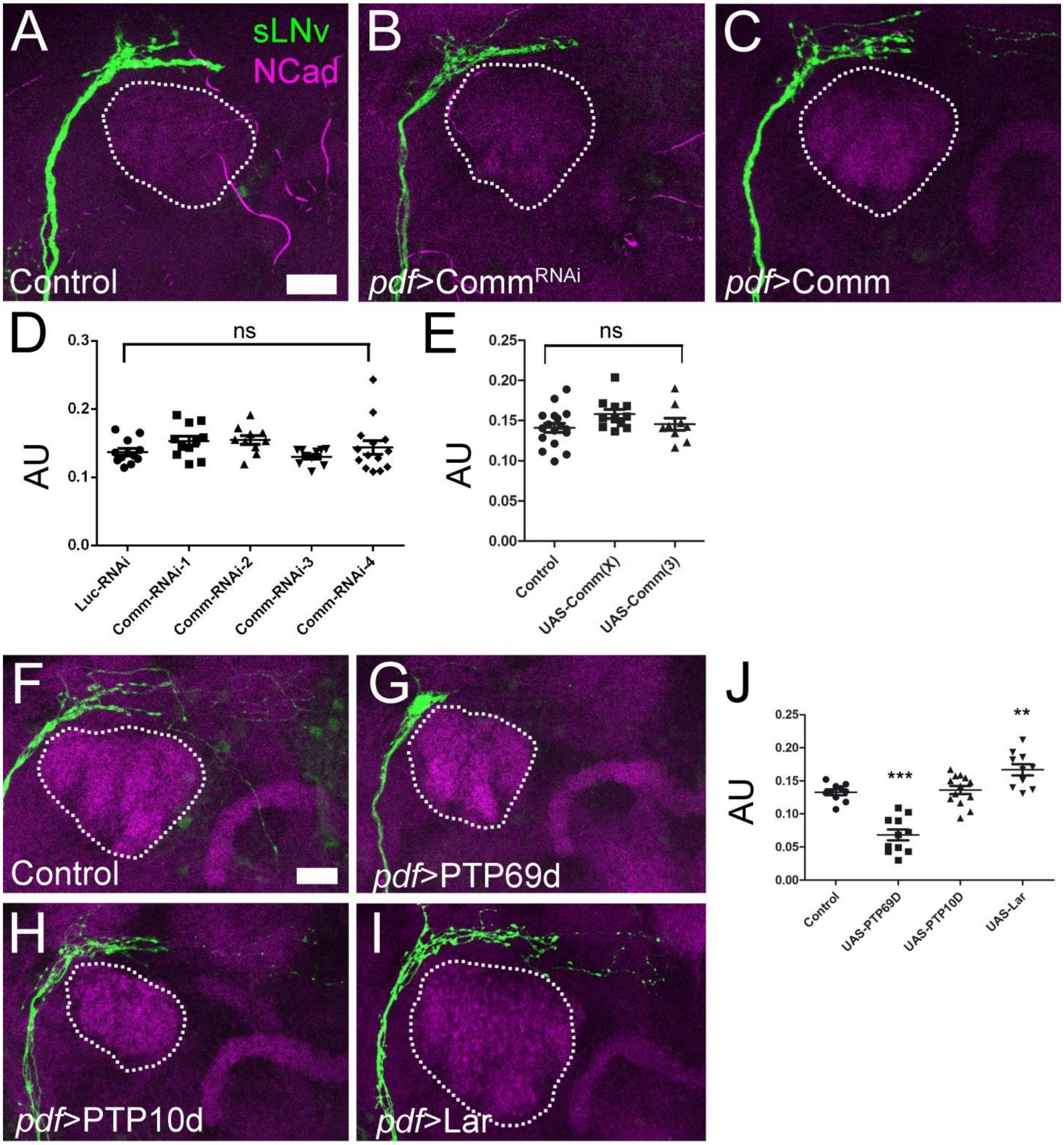

Figure S5, related to Figure 6

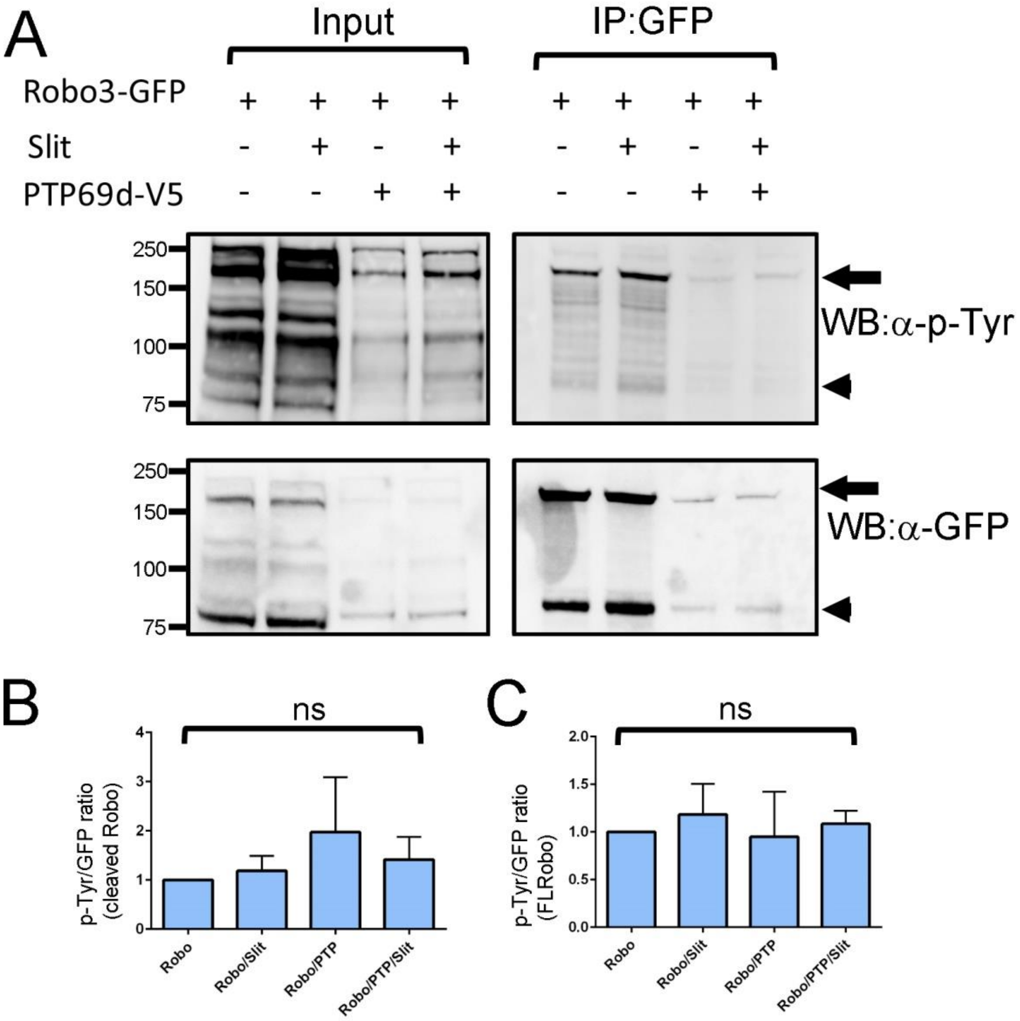

Figure S6 related to Figure 7

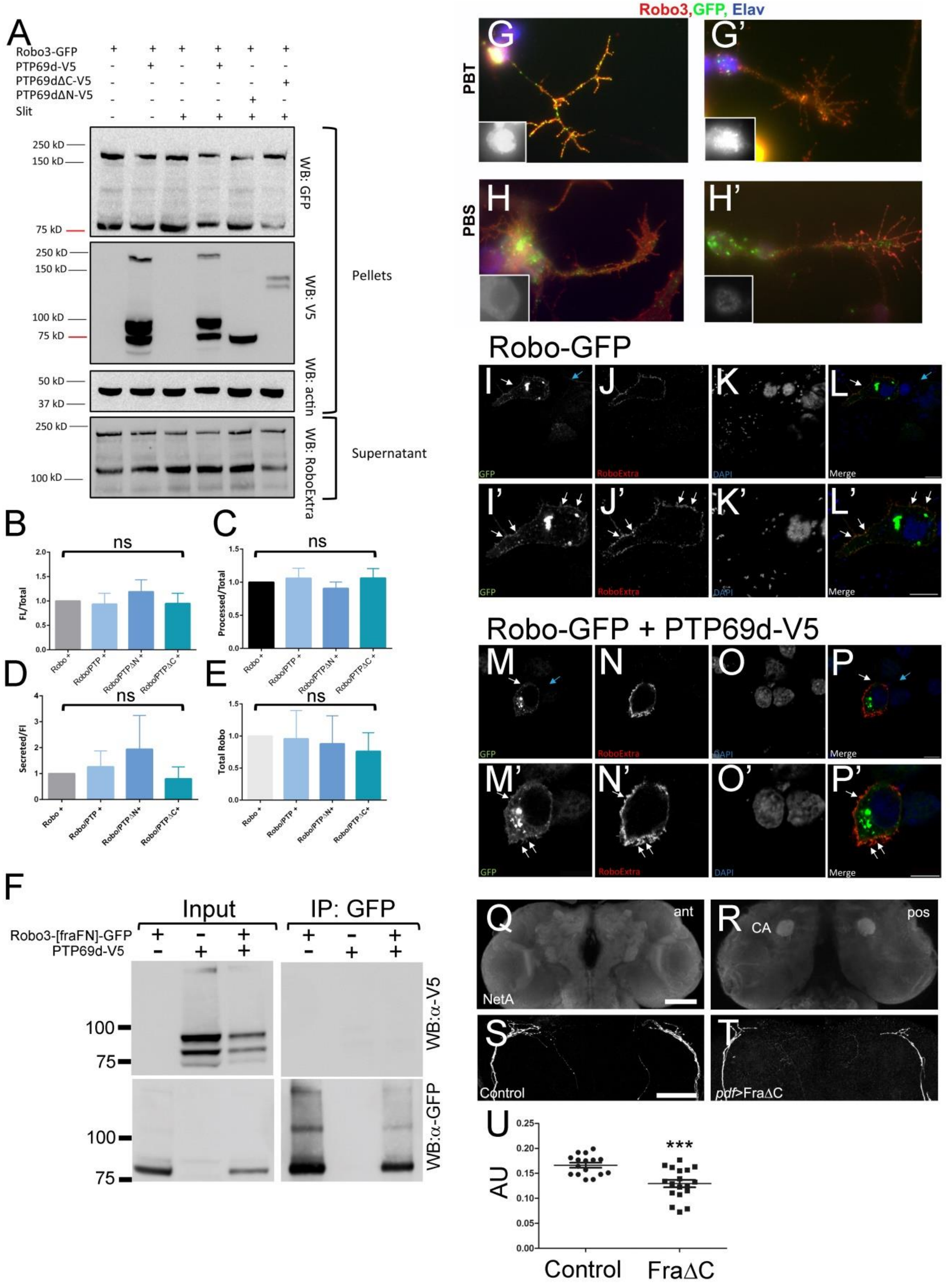

Supplemental Figure legends

FIGURE S1. Related to Figure 1. (A-K) Slit is expressed in the MB neuropile.

(A-F) Staining of larval brains with the indicated antibodies. (A-C) Slit (red) is expressed in the mushroom body neuropile, reported by GFP under the control of *OK107-Gal4* (green). (D-F) Absence of colocalization is observed with the wrapping glia marker *Nrv2-Gal4*, indicating that Slit is only in the neurons. (G, I) Staining of adult brains with Slit antibody in wild type (G), Slit knock-down (KD) in MB (H) and glial cells (I). The MB calyx is surrounded with a dashed line. (J, K) Quantification of Slit intensity (data presented as mean $\pm$ SEM, Mann-Whitney test, $**p<0.01$).

(L-W) Robo expression patterns in the central brain. (L-Q) Staining of larval brains using the indicated antibodies, mushroom area is surrounded by dashed lines. (R-W) Close up views of the panels above (single sections) in regions indicated by yellow dashed squares. Note that Robo1, Robo2 and Robo3 show no significant expression in the MBs. Scale bar: 30 μm

FIGURE S2. Related to Figure 2. (A-E'') Expression pattern of Robos and RPTP69d in sLNv.

(A- C'') Analysis of the expression pattern of different Robo proteins (red) in s-LNv axons (green) in L3 stage. (A', A'', B', B'', C', C'') Close up views (single sections or projections of 2 to 3 optical slices of 0.8 um thickness) in regions indicated with yellow dashed squares, all Robo receptors show detectable expression (arrowheads), the area of s-LNv axons is surrounded by dashed lines. (D-D'') Expression analysis of Robo3 in L3 stage, using a GFP genomically-tagged line (RMCE-MIMIC) in the green channel, PDH antibody is used to highlight pdf neurons (red). (D', D'') Close up views acquired similar to above. (E, E'') Expression analysis of RPTP69d using a specific antibody (red), sLNv axons are depicted in green. (E', E'') Close up views acquired similar to above. RPTP69d is detected in sLNv axons (arrow in E' and E'').

Expression pattern of Robos and RPTP69d in DCN axons.

(F-H'') Expression pattern of different Robo proteins (red) in the DCN axons (green) in L3 stage. (F', F'', G', G'', H', H'') Close up views (single sections or projections of 2 to 3 optical slices of 0.8 um thickness) of regions indicated with yellow dashed squares. Note that none of the Robo receptors show significant expression above background levels. (I-I'') Expression analysis of RPTP69d protein (red) in DCN axons (green) in L3 stage. (I', I'') Close up views of the regions indicated by yellow dashed squares, acquired similar to above sections.

Single cell clones of sLNv phenotypes upon Robo signaling manipulation and phenotypes of lLNv commissure.

(J-L) Single sLNv axons (adult stage) labeled using *pdf-Gal4* in combination with a flip-out cassette (see Material and Methods section). (J) Control axon expressing GFP. (K) Axon expressing GFP and Robo2ΔC transgenes. (L) Axon expressing GFP and Robo2 transgenes. Notice that axons expressing Robo2ΔC overshoot their targets while those over-expressing Robo2 arrest before reaching its normal length. (M-N) Adult brains of animals bearing *pdf-Gal4* in combination with *UAS-GFP* reporter and stained with N-Cadherin antibody. (M) Control brains (arrowhead indicates lLNv commissure and arrows sLNv axon terminals). (N) Robo2 over-expression using *pdf-Gal4*, notice that sLNv terminals are shorter but lLNv commissures are unaffected. (O-S) Robo-RNAi phenotypes in the sLNv. (O-R) Staining of adult brains, bearing *UAS-Robo-RNAi* transgenes under the control of *pdf-Gal4*. Knocking down the different Robo proteins in sLNv separately do not elicit significant over-growth phenotypes, although a tendency is observed with the Robo3-RNAi. (S) Quantification of the observed phenotypes (data presented as mean $\pm$ SEM, One-way ANOVA). AU: arbitrary units.

Scale bars: (A-I''): 60 μm, (J,L): 20μm, (M,N): 60μm, (O,R): 20μm

FIGURE S3. Related to Figure 3.

(A-C''') Robo2 over-expression phenotypes in DCNs are Slit dependent. Adult brains bearing an *ato-Gal4* driver in combination with a *UAS-CD8-GFP* reporter. (A) Control DCN axons. (A'-A''') Close up view (single section or projection of 2 to 3 sections) of a region indicated by a dashed rectangle in (A), Robo2 staining is absent (red). (B) Brain over-expressing Robo2. (B'-B''') Close up view acquired similar to above of a region in (B), Robo2 staining is observed (red). (C) Brain over-expressing Robo2 in a *slit* mutant background. (C'-C''') Close up view acquired similar to above of a region in (C), Robo2 staining is present (red) but the commissure is not affected.

(D-H) sLNv phenotypes in *robo* mutants. (D-G) Adult brains bearing a *pdf-Gal4* driver in combination with a *UAS-CD8-GFP* reporter. (D) *sli/+* heterozigotes used as a control condition. (E) $robo^{1}/robo^{BG}$ transheterozigotes. (F) $robo2^{4}/robo2^{9}$ transheterozigotes. (G) $robo3^{1}$ homozigote mutants. (H) Quantification of the observed phenotypes (data presented as mean $\pm$ SEM One-way ANOVA, $***p<0.001$).

(I-L) Early *robo2* mutant phenotypes of sLNv neurons. Adult brains of the indicated genotypes labeled with anti-PDH (green) and anti-N-Cadherin (magenta). (I) Control brain showing the sLNv wild type pattern. (J-K) Examples

of *robo2⁴/robo2⁹* showing axonal defects. (L) Example of *robo2⁴/robo2⁹* showing misplaced sLNv cell bodies (arrow).
AU: arbitrary units. Scale bars: (A, B, C): 80µm, (A'-A''', B'-B''', C'-C'''): 40µm, (D-L): 20µm.

FIGURE S4. Related to Figure 4.
(A-E) Commissureless does not act in sLNv axon growth. (A-C) Staining of adult brains bearing *UAS-Comm-RNAi* (B) or *UAS-Comm* (C) under the control of *pdf-Gal4*. Note that no effect is observed on the sLNv axon growth in any of the conditions. (D-E) Quantifications of the effects (data presented as mean ± SEM, One-way ANOVA).
(F-J) Effect of RPTPs over-expression in sLNv axon growth. (F-I) Staining of adult brains bearing transgenes *UAS-RPTP69d* (G), *UAS-RPTP10D* (H) and *UAS-LAR* (I). Note that RPTP9d over-expressing produces a decrease of the length of the sLNv axons. (J) Quantifications of the observed phenotypes (data presented as mean ± SEM, One-way ANOVA, **p<0.01, ***p<0.001). AU: arbitrary units.
Scale bars: 20 µm.

FIGURE S5. Related to Figure 6.
Slit does not affect Robo3 phosphorylation. (A) The indicated constructs were transfected in S2 cells. Robo3-GFP was IP'd from the cells extracts alone, or in the presence of PTP69d-V5 or/and Slit. IP'd fraction was tested for tyrosine phosphorylation and there were not significant changes among the conditions. (B) Quantification of tyrosine phosphorylation levels normalized by GFP signal measured for cleaved Robo3 (arrowheads) and (C) Robo full length (arrows), data presented as mean ± SD.

FIGURE S6. Related to Figure 7.
(A-E) Robo3 shedding is not affected by RPTP69d
(A) Western blot analysis of S2 cells transfected with the indicated constructs. Robo3 processing is not affected in the presence of PTP69d constructs and Slit. (B-E) Quantification of the observed changes in processing, data presented as mean ± SD.
(F) Robo3[fraFN] does not bind RPTP69d. Co-IP experiment showing that Robo3[fraFN]-GFP is not able to co-IP RPTP69d from S2 cells.
(G-H') Antibodies do not cross the cell membrane upon no-permeabilizing conditions. We tested if we were able to detect Elav protein when no detergent was used during the staining procedure. (G, G') Staining in primary *Drosophila* neurons using the indicated antibodies. GFP is detected directly without antibody. (H, H') The same staining performed above but in absence of detergent.
(I-P') Separated channels of experiments showed in Figure 7J-L. (I-L') Membrane localization of Robo3-GFP. (M-P') Membrane localization of Robo3-GFP in the presence of PTP69d.
(Q-U) NetrinA/Frazzled signaling regulates sLNv axon growth. (A-B) NetA expression in the L3 brain, (A) anterior view and (B) posterior view. Note high NetA expression in the calyx of the MB (B). (C, D) Adult brains stained with anti-PDH antibody, in (D) expression of Frazzled-ΔC (dominant negative) using *pdf-Gal4* driver. (E) Quantification of defects in the growth of sLNv axons (Data presented as mean ± SEM, two-tail T-test, ***p< 0,001). AU: arbitrary units. Scale bars: 60 µm.

Supplemental Experimental Procedures
Fly culture
Flies were cultures on standard fly food. All experiments were performed in temperature-controlled conditions at 25C or 28C (RNAi experiments). Flip-out clones were generated by a 1hr heat shock at 37C for two days during pupal development. The following fly strains were used:
(1) CS10
(2) sli^{2}/CyO (Tayler et al., 2004)
(3) sli^{dui}/CyO (Tayler et al., 2004)
(4) $rptp69d^{1}$ (Desai et al., 1996)
(5) $robo^{1}$/CyO
(6) $robo^{BG}$
(7) $robo2^{4}$/CyO
(8) $robo2^{9}$/CyO
(9) $robo3^{1}$/CyO

(10) *UAS-CD8-GFP* (BDSC)
(11) *UAS-Robo2-EGFP* (Katsuki et al., 2009)
(12) *UAS-Robo3-EGFP* (Katsuki et al., 2009)
(13) *UAS-Robo2-ΔC* (a gift from Greg Bashaw)
(14) *UAS-Robo1-ΔC* (a gift from Greg Bashaw)
(15) *UAS-Dicer2* (BDSC)
(16) *UAS-Robo-RNAi* (Tayler et al., 2004)
(17) *UAS-Robo2-RNAi* (Tayler et al., 2004)
(18) *UAS-Robo3-RNAi* (Tayler et al., 2004)
(19) *UAS-Luciferase-RNAi* (BDSC 31603)
(20) *UAS-RPTP69d-HA* (Dascenco et al., 2015)
(21) *UAS-RPTP10d*
(22) *UAS-LAR* (BDSC 9149)
(23) *UAS-RPTP69d-RNAi* (VDRC 104761)
(24) *UAS-Slit-RNAi* (BDSC 31468)
(25) *UAS-Slit-RNAi* (VDRC 38223)
(26) Df(2R)BSC482(*sli*)/CyO (BDSC)
(27) *sli*^{2}/CyO; *Slit-RNAi* (BDSC)
(28) Df(2R)BSC482(*sli*), Slit-RNAi (VDRC)/CyO
(29) *UAS-RPTP69d-ΔN-V5* (see methods)
(30) *UAS-RPTP69d-ΔC* (DGRC 109088)
(31) *UAS-RPTP69d-extracellular-V5* (see methods)
(32) *pdf-Gal4*
(33) *ato-Gal4*
(34) *elav-Gal4*
(35) *UAS-Dicer2; UAS-CD8-GFP; OK107-Gal4*
(36) *UAS-CD8-GFP; ato-Gal4, UAS-LacZ*
(37) *UAS-Dicer2; UAS-CD8-GFP/sli2; Slit-RNAi/+; OK107-Gal4*
(38) *UAS-Comm(X)* (a gift from Matthias Landgraf)
(39) *UAS-Comm(3)* (a gift from Barry Dickson)
(40) *UAS-Comm-RNAi-1* (BDSC 28381)
(41) *UAS-Comm-RNAi-2* (VDRC 5277GD)
(42) *UAS-Comm-RNAi-3* (VDRC 5278GD)
(43) *UAS-Comm-RNAi-4* (VDRC 110488KK)
(44) *UAS-Frazzled-ΔC* (a gift from Greg Bashaw)
(45) *Robo3MIMIC-GFP* (BDSC 60286)
(46) *hsFlp; UAS <CD2,Y>GFP*
(47) *GMR31A10-Gal4* (Janelia Farm)

Flybase gene references
slit: FBgn0264089; robo1: FBgn0005631; robo2: FBgn0002543; robo3: FBgn0002543; comm: FBgn0010105; Abelson: FBgn0000017 ; Ptp69D: FBgn0014007; Lar: FBgn0000464; Ptp10D: FBgn0004370; NetA: FBgn0015773; frazzled: FBgn0011592.

Cloning
cDNA for HA-tagged RPTP69D in pUAS-attB was obtained from DGRC (UFO0693). For the V5-tagged versions the full ORF except the stop-codon was PCR-amplified and TOPO cloned in frame with the V5 tag of the vector pIB-V5/HIS-TOPO (Life-Technologies).

<u>RPTP69dΔC:</u>
The N-terminal region of PTP69d (residues 1-834) including the transmembrane domain (residues 806-823) including a V5 tag was amplified by PCR and cloned into pUAST-attB vector.

<u>RPTP69dΔN:</u>
The signal peptide (residues 1-29) and the C-terminal region (residues 797-1462) including the transmembrane domain including a V5 tag were PCR amplified and fused by overlapping PCR. Then, digested with EagI-Acc65I

enzymes and ligated into pUAST-attB using NotI and Acc65I restriction sites. The construct was injected by Bestgene in an attP line to generate transgenic flies.

RPTP69dExtra:
The N-terminal region of RPTP69d lacking the transmembrane domain (residues 1-796) including a V5 tag was amplified by PCR and cloned into pUAST-attB using the same strategy as above.
Robo3-[fraFN]-GFP:
To generate a Robo3 resistant to Kuzbanian mediated cleavage as it has been described for Robo1 (Coleman et al., 2010), the fibronectin III (FNIII) domains of Robo3 were replaced by the first three Frazzed FNIII. The construct was tagged in the C-Terminal domain with EGFP and cloned in pCDNA3 using StuI restriction sites.

In situ hybridization
In situ hybridization was carried out on L3 brains fixed with 4% formaldehyde using digoxigenin-labeled antisense RNA probes according to standard protocols. To synthesize the probes we amplified a fraction of Slit from genomic DNA using the oligos GTGGGTAAAGTGGTCCATGC and AAGCTGATCCTGTCCACACC. This PCR product was cloned in pGEM-T (promega) then linearize with SpeI and transcribed from T7 promoter for antisense synthesis.

Antibody staining of *Drosophila* brains
For adult and larval brain staining. Animals were dissected in PBS and fixed in PBT 4% formaldehyde for 15 to 20 min. Fixed brains were washed three times for 15 to 20 min in PBT and incubated with the PAXDG buffer (PBT, 5% normal goat serum, 1% BSA, 0.3% deoxycholate) for 30 min to 1 hr at room temperature. Primary antibody incubation was done in PAXDG overnight at 4°C. Then the samples were washed three times with PBT and incubated with the appropriate secondary antibody in PAXDG for 2 to 4 hr, washed with PBT and mounted using the Vectashield mounting medium (Vector, Burlingame, CA, USA). The following antibodies were obtained from the Developmental Studies Hybridoma Bank (DSHB, Iowa city, IA, USA): mouse anti-Slit (1:20), mouse anti-Robo (1:50), mouse anti-Robo3 extracellular (1:50), mouse anti-PDH (1:50), mouse anti-Fasciclin2 (1:50), rat anti N-Cadherin (1:10). Other antibodies used were: rabbit anti-NetA and rabbit anti-Robo2 (gifts from Barry Dickson;1:1000 and 1:500 respectively) mouse anti-GFP 3E6 (Invitrogen, catalog number A11120, 1:250), rabbit anti-GFP (Invitrogen, Carlsbad, CA, USA, catalog number A11122, 1:500) rabbit anti-Robo2 (1:1000), rabbit anti-DsRed (Clontech, Mountain View, CA, USA, catalog number 632496; 1:500) and anti-HRP (Cy5-conjugated Jackson ImmunoResearch 1:50). Secondary antibodies conjugated with Alexa 488, Alexa 555 and Alexa 647 were obtained from Invitrogen and used at 1:500.

Antibody staining of *Drosophila* primary neuron culture
Drosophila primary neuron cultures were generated as described previously (Goncalves-Pimentel et al., 2011; Sanchez-Soriano et al., 2010). In brief, stage 11 embryos (6-7 hrs AEL at 25°C) were homogenized, treated for 5 min at 37°C with dispersion medium, washed and dissolved in Schneider's medium (Schneider, 1964). Then, the aliquots were transferred to coverslips, kept as hanging drop cultures in airtight special culture chambers for 6 hrs at 26°C. For immunocytochemistry, cells were fixed (30’ in 4% paraformaldehyde in 0.05 M phosphate buffer, pH 7.2), washed in PBS, and then incubated with antisera diluted in PBS, washed and incubated with the secondary antibody. After several washes in PBS the samples were mounted in vectashield.

Robo3 cell surface biotinylation
To quantify the levels of Robo3 at the surface we performed cell surface biotinylation assay. COS-1 cells were seeded in 10cm dishes and transfected using ViafectTM transfection reagent with 10µ of pCDNA3-Robo3-GFP, or pCDNA3-Robo3-Uncleavable-GFP in presence or absence of pUAST-RPTP69d-V5. After 48h of transfection cells were washed 4 times in PBS pH 8.0 and then subsequently incubated for 30minutes slowly rocking at 4ºC with 0,5 mg/ml Biotine. At the end of the incubation time the cells were washed again in PBS pH 8.0 and quenched for 15 min with 3 ml PBS, 100 mM Glycine, 0.5% BSA fraction V, at 4ºC on slow rocking. After a 3 washes in PBS, 100 mM Glycine the cells were scraped in 1ml of Lysis buffer (50 mM HEPES, pH 7.2, 100 mM NaCl, 1% Triton, + proteases inhibitor), passed through a 26GA3/8 needle and incubated for 20 minutes on ice. The lysates were subsequently spinned at 4ºC for 15 minutes at max speed and the concentration of the supernatant was determined using the Bradford colorimetric assay. 40µg of proteins were kept as input and diluted in Laemly Buffer, while 600µg were incubated with 35 µl of Streptavidine beads Overnight at 4ºC. The following day the beads were

washed 4 times by spinning 1 min, 4000 rpm at 4°C and resuspending in 1ml of lysis buffer. After the last was the beads were spinned at max speed for 1 minute and resuspended in 15µl of 2x Laemly buffer.
This assay was performed also in Hela cells using the same protocol but two 10cm dishes per condition.

Antibody feeding assay
HEK-293T cells were seeded in a 12 well containing Poly-D-lysing coated glass coverslips and transfected using Fugene with 1µg of pCDNA3-Robo3-GFP and pUAST-RPTP69d-V5. After 48h of transfection cells were washed once in PBS and incubated slowly rocking at 4ºC with 500µl of mouse anti-Robo-Extracellular (Hybridoma Bank) diluted 1/100 in PBS. After 1h the cells were washed 3 times in PBS and fixed in 4% PFA for 20 minutes at RT. The PFA was subsequently washed 3 times with PBS and the cells were permeabilized for 10 minutes with 0,1% Triton X-100. Upon 3 washes in PBS the cells were incubated with secondary antibody anti-mouse Alexa 555 and DAPI for 30 minutes at room temperature. Cells were finally mounted on slides with a drop of Glycergel Mounting Medium (Dako) and subsequently imaged on a Nikon confocal.

Imaging
Imaging was performed using a Leica SP5 and SP6 confocal microscopes (Wetzlar, Germany). Images were processed using the ImageJ software (National Institutes of Health, Bethesda, MD, USA). Figures were prepared using Adobe Photoshop (Adobe, San Jose, CA, USA).

Image intensity quantification
Quantification of Slit protein:
Images of adult brains stained with Slit antibody in control and Slit knock down condition were quantified using Image J. For each image, the signal was measure in the calyx and background measure in the ventral region of the brain was subtracted.
Robo3 measurements in cultured neurons:
To quantify the levels of Robo3 at the surface we use anti-Robo3 extracellular, in the absence of detergent. Surface Robo3 was quantified in FIJI (Schindelin et al., 2012), by drawing a line along the outline of axons and measuring the mean intensities within. Each mean value of surface Robo3 was divided by the mean of total overexpressed Robo3. The levels of total overexpressed Robo3 were obtained by measuring the mean intensity of GFP in FIJI as indicated above.
Antibody feeding analysis:
For analysis, the degree of colocalization was determined using Fiji/ImageJ2 macro implemented in Jython. Raw images were imported using BioFormats library. Individual cells were manually segmented in each image slice by the user. Stack threshold levels for each channel were calculated using preselected autothresholding algorithms available in Fiji (Yan for the green channel and Max Entropy for the red channel). Determined threshold levels were used to calculate Mander's overlap coefficient using ImgLib2 implementation of the colocalization algorithm. Code for the macro is available on GitHub (https://github.com/rejsmont/FijiScripts/blob/master/mColoc3D.py).

Immunoprecipitation
S2 cells were cultured in 6 well plates at 25C using in Sf900II medium. Electroporation was carried out using the Amaxa V kit (Lonza, Basel, Switzerland). The constructs used were pMT-Gal4, pUAST-GFP, pUAST-Robo3-GFP (Yasushi Hiromi), and pUAST-RPTP69d-V5. For Slit treatment, Slit was obtained from the supernatant of S2 stable-expressing cells and was added 3 days after transfection. 6 hours later cells were extracted and the pellet frozen.
Cells were lysed using 400 ul of Ripa buffer (50mM Tris pH: 7,5; Glycerol 10%; NP-40 0,4%; 150mM NaCl and fresh added protease inhibitors cocktail 100x (Amresco). NaF and Na3VO4 phosphatase inhibitors were added if detection of tyrosine phosphorylation was required) per well (from a 6 well plate). 25 ul of anti-GFP conjugated beads (Chromotek) were washed 3 times with Ripa buffer. Then, 250 ul of cell lysate was mixed the beads and the volume brought to 1 ml, and then incubated 2hrs at 4C in a rotator, followed by 4 washes with Ripa buffer 10 min each. Finally, the elution was performed with 15 ul of laemmeli Buffer (2x) followed by boiling 5 min at 95C and stored at -20C.

Western Blotting
SDS-PAGE was performed using 4-12% gradient polyacrylamide gels and then transferred to nitrocellulose membranes. For blotting, Mouse anti-V5 cat no R96025 (1:500, Invitrogen-Life Technologies) , Mouse anti-Phospho-Tyrosine clone 4G10 cat no 05-321 (Millipore), Rabbit anti-GFP cat no A6455 (1:1000, Invitrogen-Life

Technologies) , Slit antibody (1:100, DSHB, Iowa city, IA, USA) and Mouse anti-actin cat no ab3280 (Abcam) were used. Secondary antibodies were anti-rabbit HRP or anti-mouse-HRP (Jackson). Visualization of the samples was performed using ECL Western Blotting Detection System (GE Healthcare, UK). Quantifications Robo3 processing were performed by densitometry of the blots. Total Robo was determined by addition of the full length and processed form normalized by the actin loading control. Then the ratio full length v/s total was plotted.

Statistics

Statistical analysis was performed using the Prism software (GraphPad Software Inc, La Jolla, CA, USA). For sLNv measurements, results are presented as arbitrary units (A.U.) representing the fraction between the lengths of the dorsal projections divided by the distance between cell bodies in the two brain hemispheres. For analysis a two-tailed t-test was used for two-group comparisons and ANOVA for multiple comparisons. For primary neuronal culture experiments the Mann-Whitney test was used. For analysis of Robo phenotypes in DCN neurons a Fisher Exact test was performed.

Supplemental References

Desai, C.J., Gindhart, J.G., Jr., Goldstein, L.S., and Zinn, K. (1996). Receptor tyrosine phosphatases are required for motor axon guidance in the Drosophila embryo. Cell *84*, 599-609.

Goncalves-Pimentel, C., Gombos, R., Mihaly, J., Sanchez-Soriano, N., and Prokop, A. (2011). Dissecting regulatory networks of filopodia formation in a Drosophila growth cone model. PloS one *6*, e18340.

Sanchez-Soriano, N., Goncalves-Pimentel, C., Beaven, R., Haessler, U., Ofner-Ziegenfuss, L., Ballestrem, C., and Prokop, A. (2010). Drosophila growth cones: a genetically tractable platform for the analysis of axonal growth dynamics. Developmental neurobiology *70*, 58-71.

Schindelin, J., Arganda-Carreras, I., Frise, E., Kaynig, V., Longair, M., Pietzsch, T., Preibisch, S., Rueden, C., Saalfeld, S., Schmid, B.*, et al.* (2012). Fiji: an open-source platform for biological-image analysis. Nat Methods *9*, 676-682.

Schneider, I. (1964). Differentiation of Larval Drosophila Eye-Antennal Discs in Vitro. The Journal of experimental zoology *156*, 91-103.
