# Supplementary material for: Regulation of Drosophila Brain Wiring by Neuropil Interactions via a Slit-Robo-RPTP Signaling Complex
Source: Dev Cell. 2016 Oct 24;39(2):267–78. doi: 10.1016/j.devcel.2016.09.028 (PMC5084709; doi:10.1016/j.devcel.2016.09.028)
Supplement: Document S2. Article plus Supplemental Information [file mmc2.pdf]

Developmental Cell

Article

Regulation of *Drosophila* Brain Wiring by Neuropil Interactions via a Slit-Robo-RPTP Signaling Complex

Graphical Abstract

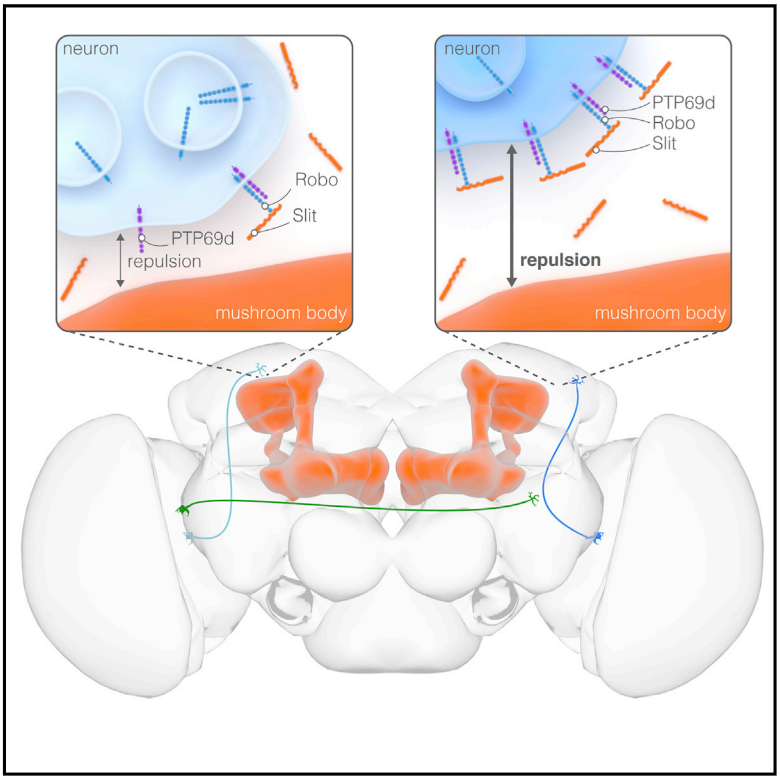

Highlights

- In the *Drosophila* brain, mushroom bodies are a source of the Slit guidance cue
- Slit regulates axon growth in the vicinity of mushroom bodies via Robo receptors
- The phosphatase RPTP69D regulates Robo signaling in the brain
- RPTP69D regulates Robo3 membrane presentation independent of its enzymatic activity

Authors

Carlos Oliva, Alessia Soldano, Natalia Mora, ..., Dietmar Schmucker, Natalia Sanchez-Soriano, Bassem A. Hassan

Correspondence

bassem.hassan@icm-institute.org

In Brief

Slit/Robo signaling is a conserved pathway regulating axon guidance. In the *Drosophila* ventral nerve cord, Robo is regulated by the protein Commissureless, but this mechanism is not conserved. Oliva, Soldano et al. show that in the *Drosophila* brain, the phosphatase RPTP69D regulates Robo membrane levels, independently of its enzymatic activity.

Oliva et al., 2016, Developmental Cell *39*, 267–278
October 24, 2016 © 2016 The Author(s). Published by Elsevier Inc.
http://dx.doi.org/10.1016/j.devcel.2016.09.028

Developmental Cell
Article

Regulation of *Drosophila* Brain Wiring by Neuropil Interactions via a Slit-Robo-RPTP Signaling Complex

Carlos Oliva,[1,2,3,8] Alessia Soldano,[1,2,8] Natalia Mora,[1,2] Natalie De Geest,[1,2] Annelies Claeys,[1,2] Maria-Luise Erfurth,[2,4,5] Jimena Sierralta,[3] Ariane Ramaekers,[1,2] Dan Dascenco,[2,4] Radoslaw K. Ejsmont,[1,2,7] Dietmar Schmucker,[2,4] Natalia Sanchez-Soriano,[6] and Bassem A. Hassan[1,2,7,9,*]

[1]Laboratory of Neurogenetics, VIB Center for the Biology of Disease, Vlaams Instituut voor Biotechnologie (VIB), 3000 Leuven, Belgium
[2]Center for Human Genetics, University of Leuven School of Medicine, 3000 Leuven, Belgium
[3]Physiology and Biophysics Department, Biomedical Neuroscience Institute, Faculty of Medicine, University of Chile, Independencia 1027, 8380453 Santiago, Chile
[4]Neuronal Wiring Laboratory, VIB Center for the Biology of Disease, Vlaams Instituut voor Biotechnologie (VIB), 3000 Leuven, Belgium
[5]Institute of Biochemistry, Christian-Albrechts-University of Kiel, 24118 Kiel, Germany
[6]Cellular and Molecular Physiology, Institute of Translational Medicine, University of Liverpool, Liverpool L69 3BX, UK
[7]Sorbonne Universités, UPMC Univ Paris 06, Inserm, CNRS, Institut du cerveau et la moelle (ICM) - Hôpital Pitié-Salpêtrière, Boulevard de l'hôpital, 75013 Paris, France
[8]Co-first author
[9]Lead Contact
*Correspondence: bassem.hassan@icm-institute.org
http://dx.doi.org/10.1016/j.devcel.2016.09.028

SUMMARY

The axonal wiring molecule Slit and its Round-About (Robo) receptors are conserved regulators of nerve cord patterning. Robo receptors also contribute to wiring brain circuits. Whether molecular mechanisms regulating these signals are modified to fit more complex brain wiring processes is unclear. We investigated the role of Slit and Robo receptors in wiring *Drosophila* higher-order brain circuits and identified differences in the cellular and molecular mechanisms of Robo/Slit function. First, we find that signaling by Robo receptors in the brain is regulated by the Receptor Protein Tyrosine Phosphatase RPTP69d. RPTP69d increases membrane availability of Robo3 without affecting its phosphorylation state. Second, we detect no midline localization of Slit during brain development. Instead, Slit is enriched in the mushroom body, a neuronal structure covering large areas of the brain. Thus, a divergent molecular mechanism regulates neuronal circuit wiring in the *Drosophila* brain, partly in response to signals from the mushroom body.

INTRODUCTION

During nervous system development, proper axon guidance is achieved through the interaction between neuronal cell surface receptors and their chemoattractive or repulsive ligands present in the environment (Chilton, 2006; Dickson and Gilestro, 2006; Lowery and Van Vactor, 2009). The Slit/Robo signaling pathway (Dickson and Gilestro, 2006) plays essential functions during axon pathfinding in many neural populations, and special attention has been given to its role in commissural axon development (Dickson and Gilestro, 2006). Upon Slit binding to its receptor Robo, axons are repelled from the Slit source in most systems (Dickson and Gilestro, 2006). The *Drosophila* embryonic ventral nerve cord (VNC) has served as a powerful model system for the study of axon guidance by the Slit/Robo pathway (Dickson and Gilestro, 2006) whereby glial cells along the midline express Slit, which acts as a repulsive cue to guide neuronal axons toward or away from the midline in function of their repertoire of Robo receptors. This is similar to mammalian spinal cord where a specialized midline structure called the floor plate acts as a major source of guidance cues (Chedotal, 2011). In *Drosophila* there are three Robo receptors (Robo1, Robo2, and Robo3) and one Slit ligand. Robo1 and Robo2 are involved in commissure formation in the embryo while Robo 2 and Robo 3 regulate the formation of ipsilateral pathways (Rajagopalan et al., 2000). In contrast to the VNC, it is less clear how axon guidance is organized in higher-order brain centers. Roles for Slit and/or Robo receptors have been established in guiding peripheral axons to the brain (Jhaveri et al., 2004; Pappu et al., 2011) and Robo loss-of-function mutants, or pan-neuronal downregulation, causes broad defects (Nicolas and Preat, 2005; Tayler et al., 2004), suggesting a potentially important role for this ligand-receptor pair in adult brain connectivity. In the *Drosophila* VNC a major mechanism of regulating Robo activity is via the protein Commissureless (Comm) (Keleman et al., 2002, 2005; Tear et al., 1996), which binds Robo receptors and negatively regulates their activity. Comm has not been identified in other taxa, however, suggesting that this mechanism is unlikely to be conserved.

Receptor protein tyrosine phosphatases (RPTP) belong to a family of transmembrane proteins that are characterized by three extracellular immunoglobulin (Ig) domains and usually four to eight FNIII repeats, resembling adhesion molecules such as N-CAM (Chagnon et al., 2004; Siu et al., 2007), and two tandem

This is an open access article under the CC BY license (http://creativecommons.org/licenses/by/4.0/).

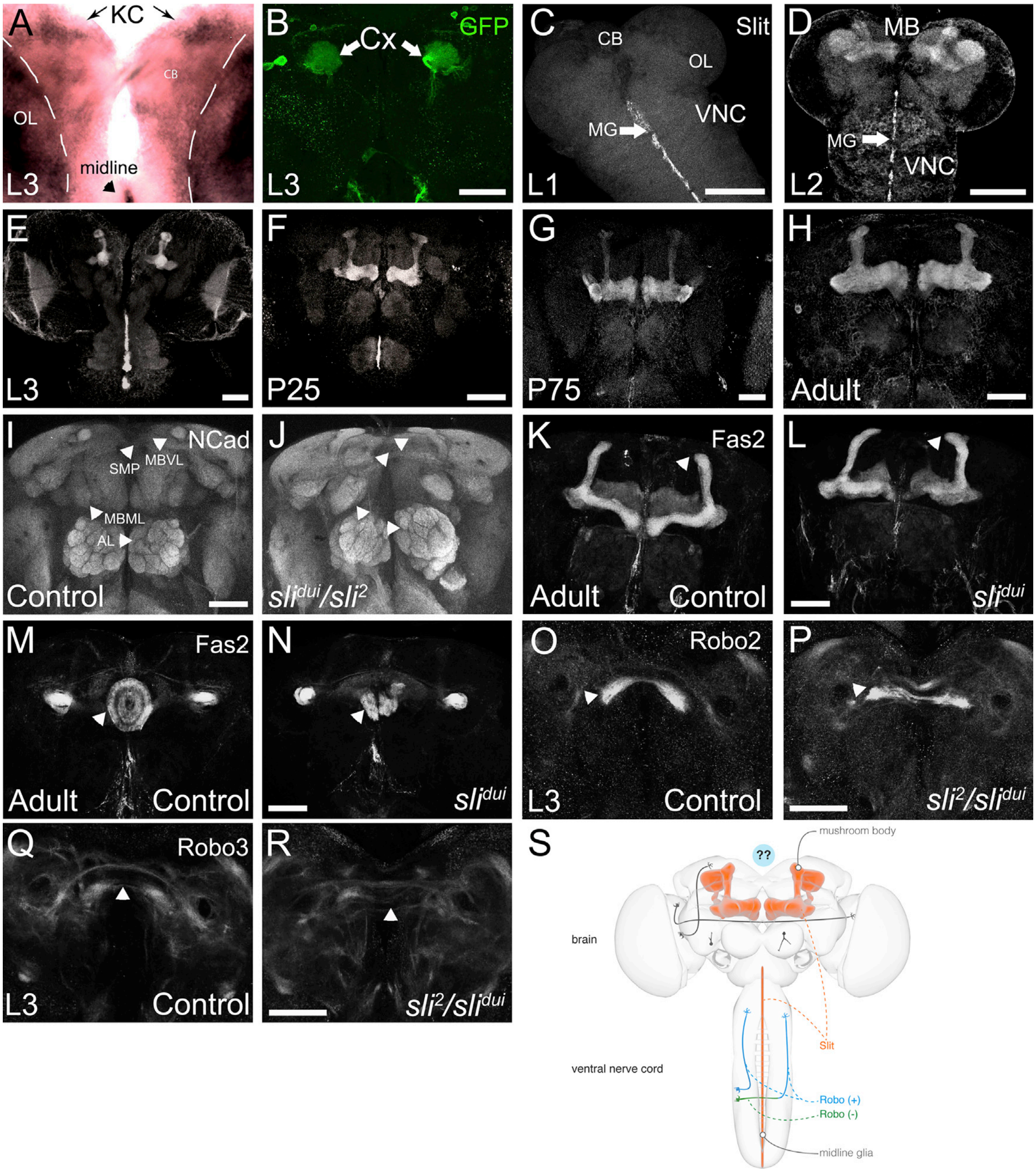

Figure 1. Slit Expression Pattern and Phenotypes during Brain Development

(A) Slit RNA expression in the larval brain using in situ hybridization. KC, Kenyon cells; OL, optic lobe; CB, central brain.

(B) *slit* enhancer (*GMR31A10-Gal4*) driving GFP shows expression in MB. Cx, mushroom body calyx.

(C) Slit expression in L1 stage; larval CNS was stained with Slit (gray). Note that Slit is not expressed in any structure in the brain at this stage while strong expression is seen in the ventral midline glia (MG). VNC, ventral nerve cord; OL, optic lobe; CB, central brain.

(D–H) Slit expression from L2 until adult stage. Starting at L2 Slit is enriched in the mushroom bodies (MB). Note that the expression in the VNC disappears at 75 hr after puparium formation but Slit continues to be expressed until adult stage in MB.

(I) The wild-type pattern of the neuropil in the adult brain revealed with anti-N-cadherin. MBVL, mushroom body vertical lobe; SMP, superior medial protocerebrum; MBML, mushroom body medial lobe; AL, antennal lobe.

(J) Pattern of the neuropil in the adult brain revealed with anti-N-cadherin in *slit* mutants presents widespread and strong defects in neuropil architecture (arrowheads).

(K–N) Mushroom body and ellipsoid body architectures revealed with anti-Fas2 in wild-type animals (K, M) and *slit*^{dui} mutants (L, N). Arrowheads indicate specific brain structures (mushroom body α-lobes in K and L), ellipsoid body in M and N).

(O–R) Pattern of Robo2- and Robo3-expressing axons in L3 stage in normal and *slit* mutant animals. Note that in wild-type brains Robo2- and Robo3-positive commissures are present (O and Q, see arrowheads). In *slit* mutant brains the integrity of these commissures is disturbed (P and R, see arrowheads).

(legend continued on next page)

intracellular catalytic domains (D1 and D2) with putative phosphatase activity. RPTPs have been shown to play an important role in nervous system development. In both flies and vertebrates, RPTPs play a role in guidance of motor axons (Stepanek et al., 2005). In the fly embryonic nervous system, RPTP69d and RPTP10d have been shown to regulate commissure development and to genetically interact with the Slit/Robo pathway (Sun et al., 2000). Based on the presence of phosphatase domains in these proteins, it has been speculated that RPTP69d and RPTP10d activate Robo by dephosphorylating it. However, this assumption has not been experimentally tested in any model.

Here we show that the higher-order *Drosophila* brain region, known as the protocerebrum, does not contain midline sources for the major axon growth and guidance cue Slit. Instead, the mushroom body (MB) is the major source of Slit in the developing protocerebrum. The MB is a large, highly conserved, insect neuropil composed of the axons and dendrites of approximately 2,500 neurons called the Kenyon cells. The MB is required for associative learning and memory, as well a host of innate and learned behaviors (Heisenberg et al., 1985; Krashes et al., 2007; Pitman et al., 2006). We show that Slit expression within the MB is essential for the correct patterning of neighboring higher-order neural circuits. Specifically, the interaction between Robo receptors and RPTP69d is necessary and sufficient for repulsive axonal responses to Slit from the MB. While RPTP69d co-expression enhances the effect of both Robo receptors, it has no repulsive or Slit binding activity on its own. Surprisingly, the RPTP69d phosphatase domain is dispensable for both Robo receptor binding and axon repulsion. Instead, we find that RPTP69d enhances the cell surface presentation of Robo receptors. We propose that the MB acts as a spatially distributed neuronal source of Slit for *Drosophila* brain connectivity, which could contribute to the higher level of complexity observed in the brain compared with the VNC. Furthermore, we identify a different Slit-Robo-RPTP signaling mechanism acting in the brain.

RESULTS

Slit Is Expressed in the Mushroom Body and Is Required for Central Brain Connectivity

To explore how central brain connectivity is organized, we examined Slit and Robo expression during brain development. Visualization of Slit RNA by in situ hybridization reveals its known pattern in the VNC and optic lobe (Figure 1A). Moreover, signal is observed in two bilateral clusters consistent with the localization of MB Kenyon cells (Figure 1A). Furthermore, an enhancer fragment from the Slit locus reports strong GFP expression in the MB (Figure 1B). Next, we explored the expression pattern of Slit protein. We find that starting at the second larval instar and throughout pupal brain development, Slit protein is strongly expressed in the MB (Figures 1C–1H). Slit is expressed in both the axons and dendrites of MB neurons themselves and not in the glial scaffold that surrounds them (Figure S1). Protein localization in MB is confirmed by MB-specific knockdown of Slit showing a decreased signal in MB, in contrast to glial-specific knockdown (Figure S1). The MB is a large and spatially distributed neuropil structure, whose axons and dendrites span a significant expanse of the developing brain in all three axes (Figure 1S). All three Robo receptors are expressed in the brain in many different axonal tracts, but are specifically absent from the MB at the third instar larval stage (Figures S1L–S1W). While *slit* null mutant animals die at embryonic or early larval stages, we were able to examine the neuropil structure of viable *slit* mutants bearing a combination (sli^2/sli^{dui}) of a null allele (sli^2) and a hypomorphic allele (sli^{dui}) (Tayler et al., 2004) exhibiting a strong reduction of Slit in the larval and pupal nervous system (Dascenco et al., 2015). We found major defects in neuropil organization in several central brain areas, including MB lobes, the central complex, antennal lobes, and Robo2/Robo3-expressing axons in the developing brain (Figures 1I–1R). In summary, reduction of Slit activity in the brain causes widespread disturbances in brain neuropil architecture. Whereas in the VNC midline glia acts as a point source of a Slit gradient, in the protocerebrum the axons and dendrites of bilateral MBs are a neuronal source of broadly distributed Slit during brain development (Figure 1S).

The Mushroom Body Regulates Axon Growth and Guidance via Slit/Robo Signaling

To uncover the specific mechanisms of Slit function in the MB, we chose two neuronal populations which express or lack the Robo receptors, respectively. The circadian clock neurons, called small lateral neurons (sLNv) (Helfrich-Forster et al., 2007), express all three Robo receptors and localize them to axons (Figures S2A–S2D″). In contrast, the dorsal cluster neurons (DCNs), which are higher-order contralateral projecting neurons with axons that innervate the optic lobes (Langen et al., 2013; Zschatzsch et al., 2014) do not express Robo1–3 (Figures S2F–S2H″). The four to five sLNv axons project dorsally and then turn medially close to the MB dendritic tree known as the calyx (Figures 2A and 2B). In *slit* mutants, the medial projections extend significantly further than in control brains (Figures 2C–2E), suggesting that normally Slit limits the growth of sLNv axons. To test whether this effect is dependent on Robo receptors, we used the Gal4/UAS system to inhibit the activity of all Robo receptors specifically in the sLNv using the LNv-specific *pdf-Gal4* driver and a dominant negative Robo2 transgene, which lacks the intracellular domain (Robo2ΔC) known to inhibit all three receptors (Bashaw et al., 2000; Godenschwege et al., 2002; Kraut and Zinn, 2004). This results in a significant increase in sLNv axon length (Figures 2F–2H). Similar results were obtained with a dominant negative Robo1 transgene, which also inhibits all three receptors (data not shown). Single-cell visualization, using flip-out clones, confirmed that sLNv individual axons overshoot their targets (Figures S2J and S2K). RNAi knockdown

(S) Schematic representation of Slit responses in the VNC compared with the brain. In the VNC Slit spreads from a point source, the midline glia, whereas in the brain expression of Slit in MB generates a distributed source reaching large aspects of the developing and adult brain. Blue indicates the presence and green the absence of Robo. Gray axons indicate unknown Robo expression status, and question marks indicate lack of knowledge of how Robo-Slit signaling regulates brain wiring.
Scale bar in (B) represents 50 μm; all other scale bars represent 60 μm. See also Figure S1.

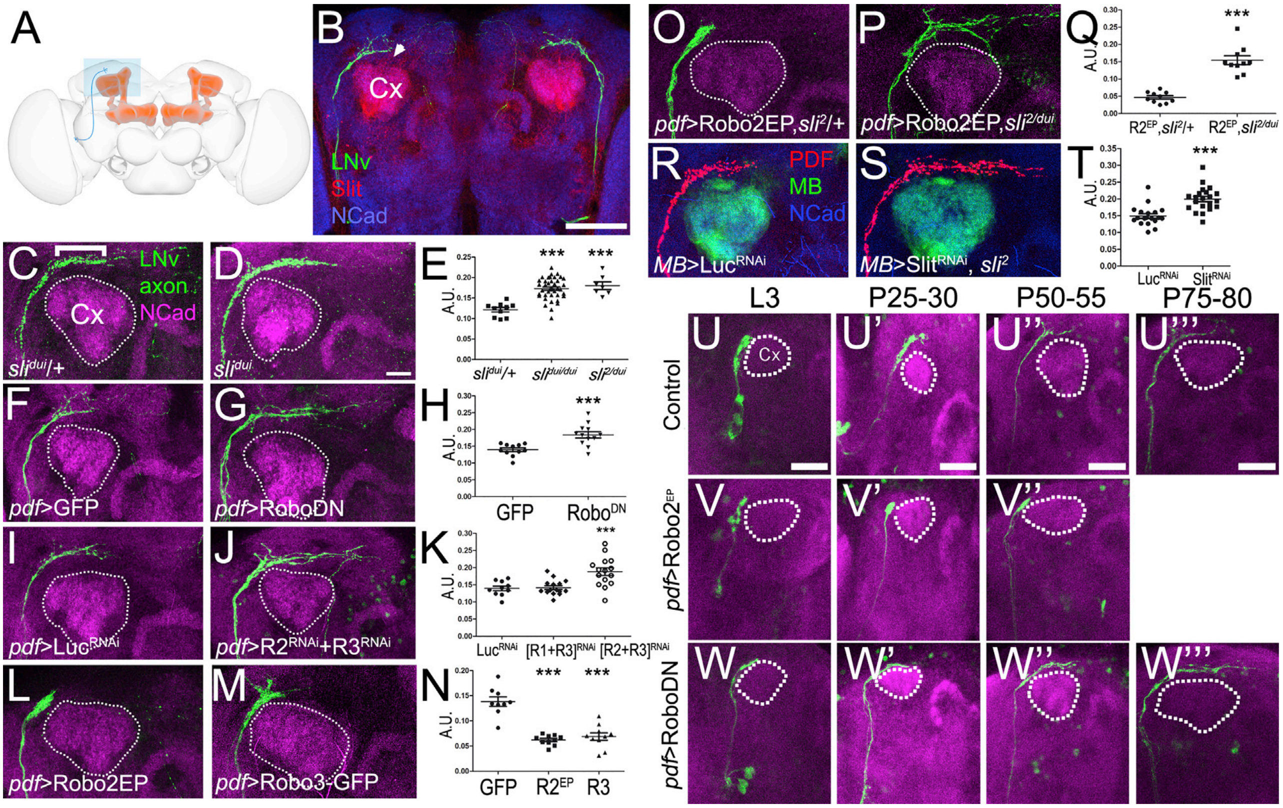

Figure 2. Changes in Neuronal Wiring upon Manipulation of Robo/Slit Signaling

(A) Schematic of the *Drosophila* brain highlighting sLNv (blue) and the MB (red).

(B) Adult brain of animals expressing GFP in LNv neurons stained with Slit and N-cadherin antibodies. Cx, MB calyx. Scale bar represents 60 μm.

(C–E) Effects of *slit* mutant on sLNv projections. (C) Control $sli^{dui/+}$ brain stained with PDH antibody (green) to label LNvs, and N-cadherin (magenta) to label the neuropil. The MB calyx (Cx) is easily distinguishable and is indicated by the dotted line. (D) sli^{dui}/sli^{dui} and sli^{2}/sli^{dui} mutants display overgrowth of the medial projection. (E) Quantification of sLNv length in the different genotypes. *slit* mutants have significantly longer medial projections than controls (***$p < 0.001$, two-tailed t test).

(F–H) Effects of loss of function of Robo receptors on sLNv projections. (F) Control brains of flies bearing *pdf-Gal4*, *UAS-GFP*, or (G) expressing a dominant negative form of the Robo receptor. (H) Quantification of the phenotypes in F and G (***$p < 0.001$, two-tailed t test).

(I–K) Effect of knocking down Robo receptors on sLNv projections. (I) *UAS-Luc-RNAi* (luciferase) was used as a control line. (J) *UAS-Robo2-RNAi* + *UAS-Robo3-RNAi*. (K) Quantification of sLNv length in (I) and (J) and Robo1/Robo3 double RNAi condition (***$p < 0.001$, two-tailed t test).

(L–N) Effects of overexpressing Robo receptors in the sLNv projections. (L) Animals expressing *UAS-Robo2EP* under the control of the *pdf-Gal4* driver. (M) Animals expressing *UAS-Robo3*. In both cases sLNv have shorter axons. (N) Quantification of the phenotypes in (L) and (M) (***$p < 0.001$, two-tailed t test).

(O–Q) The Robo gain-of-function phenotype depends on Slit. (O) Animals overexpressing Robo2 in a sli^{2} heterozygous background. (P) Overexpression of Robo2 in the *slit* mutant background (sli^{dui}/sli^{2}). Robo expression does not shorten axonal length in the absence of Slit. (Q) Quantification of the phenotypes in (O) and (P) (***$p < 0.001$, two-tailed t test).

(R–T) Effect of Slit knockdown in the MB on the growth of sLNv axons. (R) Control animals bearing the genotype *UAS-DCR2/+;UAS-CD8-GFP/+;OK107-Gal4/+*. (S) Knockdown in the MB using flies bearing the genotype *UAS-DCR2/+;*sli^{2}*/UAS-CD8-GFP/;UAS-Slit-RNAi/+;OK107-Gal4/+*. (T) Quantification of the phenotypes in (R) and (S) (***$p < 0.001$, two-tailed t test). A.U. (arbitrary units) represent the fraction between the lengths of the sLNv dorsal projections divided by the distance between cell bodies in the two brain hemispheres.

(U–W‴) Developmental analysis of sLNv axon growth in wild-type and after Robo manipulations. Brains of animals expressing GFP under the control of *pdf-Gal4* driver, obtained at different developmental stages; MB calyx is delineated by a dashed line. Anti-GFP and anti-N-cadherin (magenta) antibodies were used for labeling. (U–U‴) Wild-type development: sLNv axons grow from L3 to late pupal stages where they reach their final pattern. (V–V″) Upon overexpression of Robo2, axons get arrested when they reach the MB calyx. (W–W‴) Upon loss of function of Robo using a Robo2ΔC construct, axons grow initially as controls but overshoot in later stages.

In all column scatter plots, data are presented as mean ± SEM.

Scale bar represents 20 μm in (D) (applies to C–T) and 40 μm in (U)–(U‴) (applies to U–W‴). See also Figure S2.

of each single Robo receptor alone, or the combination of Robo1/2 knockdown, did not significantly alter the length of sLNv axons, although knockdown of Robo3 alone did show a moderate tendency toward longer axons (Figures S2O–S2S). Knockdown of the Robo2/3 combination significantly increased sLNv axonal length (Figures 2I–2K). Conversely, overexpression of either Robo2 (Rajagopalan et al., 2000) or Robo3-GFP fusion protein, which has been shown to express in a manner comparable with endogenous Robo3 (Katsuki et al., 2009; Trunova et al., 2011), was sufficient to significantly decrease sLNv axonal

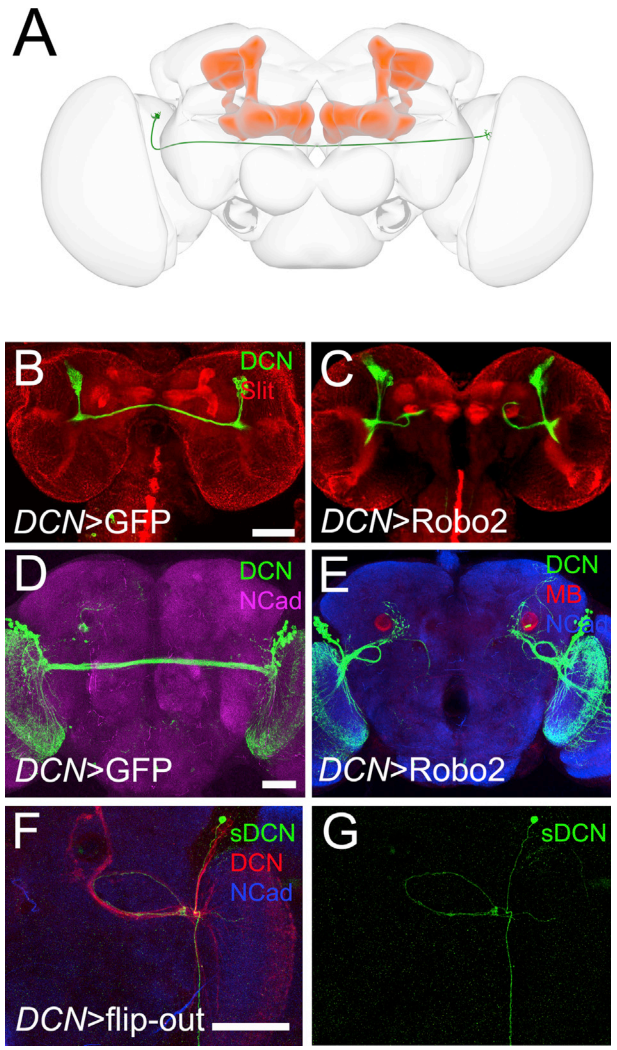

Figure 3. Robo Gain-of-Function Phenotypes in Dorsal Cluster Neurons

(A) Schematic showing dorsal cluster neurons (DCN; green) in the context of the MB (red).

(B and C) Wild-type (B) and Robo2-overexpressing animals (C) at L3. Upon Robo2 overexpression, DCN axons (green) do not form the characteristic commissure and instead stall in the proximity of the Slit-expressing MB peduncle (red).

(D and E) Adult brain of controls (D) and animals expressing Robo2EP in DCNs (E). Most commissural axons loop around at the level of the MB peduncle and target the ipsilateral optic lobe.

(F and G) Single-cell DCN clone (sDCN) using a flip-out cassette with Robo2 overexpression.

Scale bars represent 60 μm. See also Figure S3.

growth toward the MB calyx (Figures 2L–2N). Single-cell clones show that sLNv terminal branches arrest and do not grow like wild-type axons (Figures S2J and S2L). Therefore, Robo receptor signaling regulates sLNv axonal growth and guidance and Robo3 appears to exert the strongest effect on sLNv axonal repulsion. The *pdf-Gal4* driver also labels the lLNv neurons, which however project axons far from the MB in a more ventral aspect of the brain (Figure S2M). lLNv axons are unaffected by the Robo1–3 manipulations described above, indicating that Robo receptor levels and distance from the MB together determine axonal responses to Slit (Figure S2N). To test this idea, we overexpressed Robo2 in the LNv in a *slit* mutant (sli^2/sli^{dui}) background. Reduction in Slit function resulted in a full suppression of the Robo2 gain-of-function phenotype in sLNv axonal growth (Figures 2O–2Q). Finally, RNAi knockdown of *Slit* specifically in the MB in *slit* heterozygous animals (Figures 2R–2T) was sufficient to induce increased sLNv axon growth similar to that seen in *slit* mutants. To reveal whether these defects are due to overgrowth or lack of retraction during development, we performed a developmental analysis of sLNvs in wild-type, Robo2ΔC, and Robo2 overexpressing conditions (Figures 2U–2W‴). We observe that axons do not overshoot and then retract during development in wild-type animals, indicating that sLNv axons grow until they reach their final target. When Robo receptor activity is inhibited, sLNv axons overgrow their normal target area. In contrast, these axons arrest early during development under Robo receptor gain-of-function conditions.

In contrast to the sLNv, the DCN axons do project in the vicinity of the MB and appear not to be repelled by Slit (Figures 3A and 3B). However, overexpression of Robo2 in the DCNs does result in the failure of contralateral projections in 75% of the brains examined, showing that expression of Robo(s) in DCNs is sufficient to make them respond to Slit. DCN axons arrest precisely at the level of the MB in larval brains (Figures 3B and 3C) and eventually turn around and innervate the ipsilateral optic lobes in adult brains (Figures 3D–3G), although some axons stay in the vicinity of the MB, indicating that a few of them may be attracted. This phenotype is rescued in the *slit* mutant background (Figures S3A–S3C‴). This result is consistent with the lack of Robo1–3 expression observed in DCNs (Figures S2F–S2H″). Together, these data suggest that axons respond to Slit in the MB in function of (1) their distance from the MB and (2) the composition of Robo1–3 expression. Next, we analyzed the architecture of sLNv axons in *robo1*, *2*, and *3* mutants (Figures S3D–S3H). *Robo1* mutants show normal axon lengths, while *robo2* mutants display a variety of early sLNv axonal defects with variable penetrance (Figures S3I–S3L). However, the few *robo2* mutant axons that do reach the calyx display normal length of the terminal branches (Figure S3F). Only *robo3* mutants phenocopy the *slit* mutant phenotypes (Figure S3G). Altogether, these data indicate that in the sLNv all three Robo receptors play some role in regulating axonal growth, with Robo3 being the main receptor normally necessary for regulating the length and guidance of the terminal axonal arbors.

Slit Ligand and *Robo* Receptors Interact Genetically with *RPTP69d*

We sought to gain mechanistic insight into the regulation of Robo activity in central brain development. A major mechanism by which Slit/Robo signaling is regulated in the *Drosophila* VNC is negative regulation of Robo(s) by the Comm protein (Keleman et al., 2002, 2005). However, neither overexpression nor knockdown (using four different RNAi lines) of Comm in sLNv had any effects on their axonal projection (Figures S4A–S4E). Another mechanism involves the inhibition of Robo by phosphorylation of a highly conserved intracellular tyrosine (Bashaw et al., 2000). Furthermore, Robo1 was shown to genetically interact

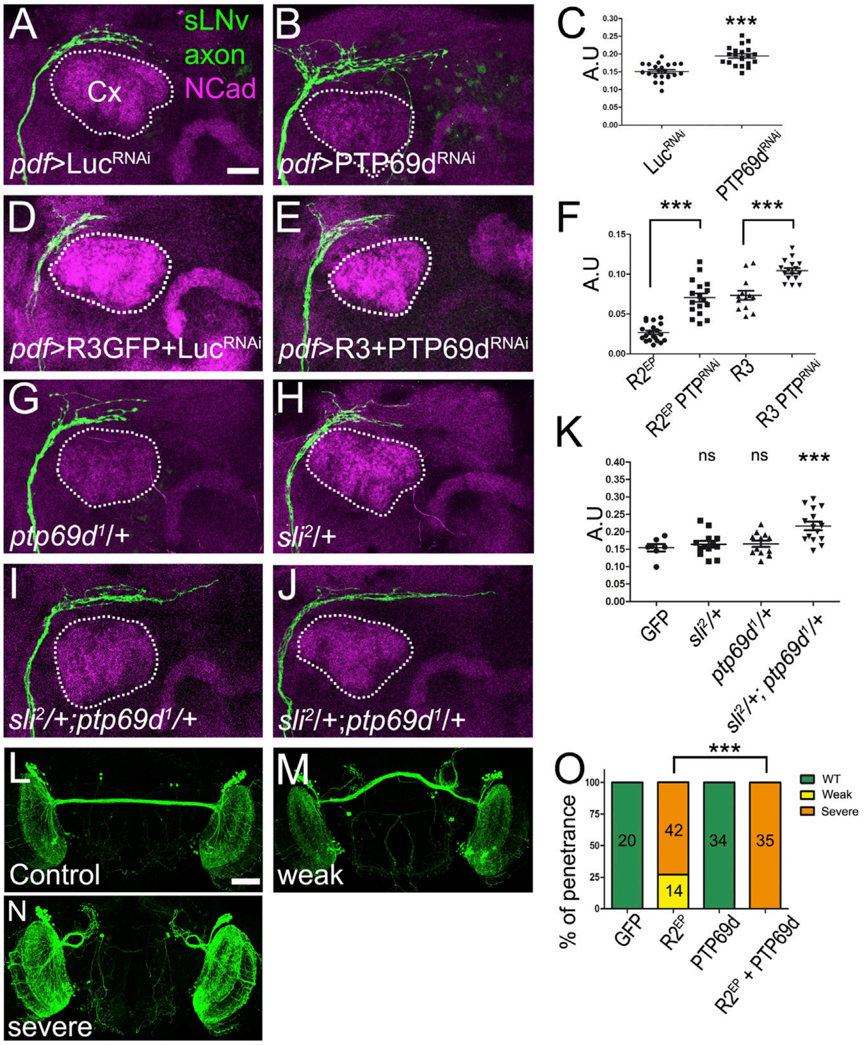

Figure 4. RPTP69d Regulates Robo Function during Axonal Growth

(A–C) Effect of knocking down RPTP69d in the sLNv projection using *pdf-Gal4* and *UAS-RPTP69d-RNAi* (B). *UAS-Luc-RNAi* is used as a control (A). Note that overgrowth of sLNv projections resembles Robo loss-of-function phenotypes. (C) Quantification of RPTP69d-RNAi effect on the projection length (***$p < 0.001$, two-tailed t test).

(D–F) Downregulation of RPTP69d partially rescue Robo2 and Robo3 overexpression phenotype. Control flies (D) with *pdf-Gal-4*, *UAS-GFP*;*UAS-Robo3-GFP*/+;*UAS-Luc-RNAi*/+ and (E) flies with reduced RPTP69d in the Robo3-GFP background (*pdf-Gal-4*, *UAS-GFP*;*UAS-Robo3-GFP*/+;*UAS-RPTP69d-RNAi*/+). (F) Quantification of the effect of RPTP69d knockdown in the Robo2 and Robo3 gain-of-function phenotype (***$p < 0.001$, two-tailed t test).

(G–K) Genetic interactions between the *ptp69d* and *slit* genes. Heterozygous flies (*pdf-Gal-4*, *UAS-GFP*;*sli*^{2}/+ and *pdf-Gal4*, *UAS-GFP*;; *ptp69d*^{1}/+) (G and H) and double heterozygotes (*pdf-Gal4*, *UAS-GFP*;*sli*^{2}/+;*ptp69d*^{1}/+) (I and J). Note that double heterozygotes show axonal overgrowth while relative to heterozygous controls. (K) Quantification of multiple animals (***$p < 0.001$, one-way ANOVA).

(L–O) RPTP69d cooperates with Robo2. (L) Wild-type, (M) weak commissure phenotype, and (N) severe commissure phenotype. (O) Quantification of the phenotypes of the different groups (***$p < 0.001$, Fisher's exact test); number of samples is indicated. Note that although expression of RPTP69d by itself does not produce defects, it enhances the Robo2 phenotype when they are expressed together. In all column scatter plots, data are presented as mean + SEM. A.U, arbitrary units.

Scale bars represent 20 μm in (A) (applies to A–J) and 60 μm in (L) (applies to L–N). See also Figure S4.

with four RPTPs (Sun et al., 2000), although no direct Robo dephosphorylation has been demonstrated. To test whether RPTPs play a role in MB-mediated Slit/Robo signaling in the brain, we overexpressed three RPTPs previously shown to regulate axon guidance (RPTP10d, RPTP69d, and Lar [Clandinin et al., 2001; Sun et al., 2000]) in sLNv. We find that overexpression of RPTP69d, but not Lar or RPTP10d, phenocopies the gain of function of Robo receptors (Figures S4F–S4J), causing premature sLNv axon arrest. In contrast, Lar leads to a moderate increase in the length of the axons, indicating that different RPTPs can have even opposite functions in axon growth.

Based on this, we studied the role of RPTP69d in sLNv axon growth and its potential interactions with Slit and Robo. RPTP69d is widely expressed in the larval brain including sLNv and DCNs (Figures S2E–S2E″ and S2I–S2I″). LNv-specific knockdown of RPTP69d by RNAi significantly increases the length of sLNv axons (Figures 4A–4C). Next, we tested whether RPTP69d interacts with the Slit/Robo pathway. Co-expression of Robo2 or Robo3-GFP with RPTP69d-RNAi partially suppresses Robo2/3 gain of function (Figures 4D–4F). Furthermore, we find that Slit and RPTP69d act synergistically as brain double heterozygous for *slit* (*sli*^{2}), and *RPTP69d* (*rptp69d*^{1}) show a significant increase in sLNv axon length compared with heterozygous controls (Figures 4G–4K). These data suggest that RPTP69d acts in the Slit/Robo pathway to regulate sLNv axonal growth.

RPTP69d gain of function stunts sLNv axonal growth and the sLNv express and require all three Robo receptors. We wanted to ascertain whether RPTP69d can induce axonal repulsion in the absence of Robo receptors. To this end, we tested RPTP69d overexpression in DCN axons, which do not express any of the three Robo receptors. RPTP69d overexpression alone in the DCNs has no effect on axonal projection, nor does RPTP69d knockdown (data not shown). In contrast, the expression of Robo2 alone causes disruption of the DCN axon repulsion at the level of the MB axonal lobes in 75% of the brains examined. When Robo2 and RPTP69d are co-expressed, the penetrance of DCN axon repulsion loss increases from 75% to 100% (Figures 4L–4O). This suggests that RPTP69d acts synergistically with Robo receptors to enhance axonal responses to Slit.

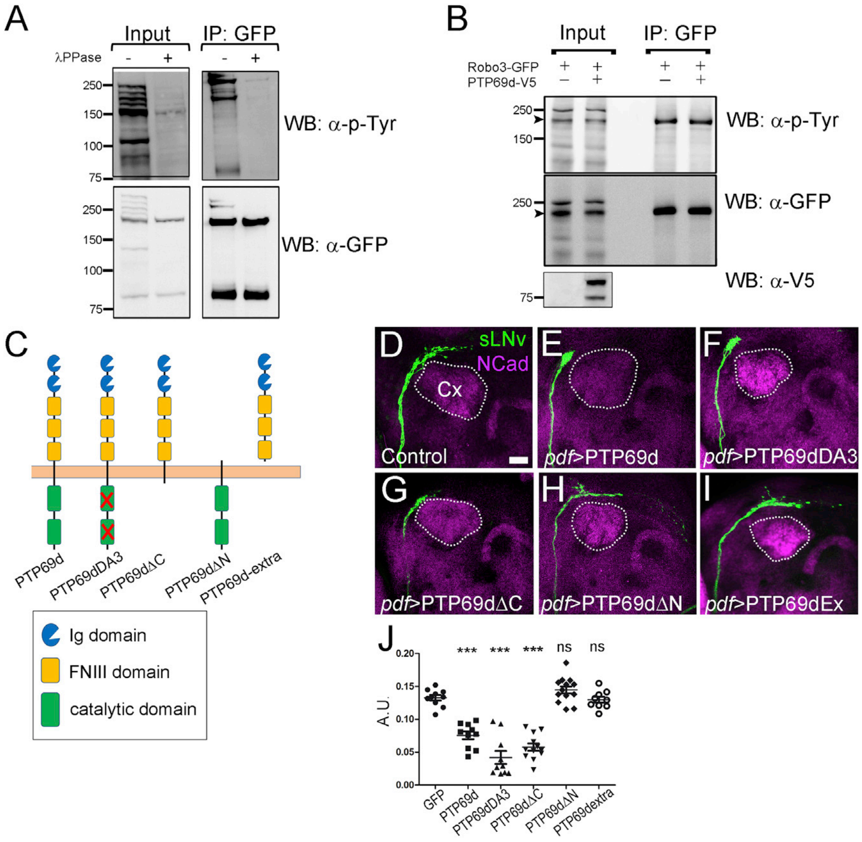

Figure 5. RPTP69d Regulates Robo Signaling Independently of Its Phosphatase Activity

(A) Western blot (WB) analysis of Robo3 tyrosine phosphorylation. Robo3-GFP-expressing S2 cells were lysed and the extract immunoprecipitated (IP) with anti-GFP and proved for tyrosine phosphorylation; λ-phosphatase treatment confirmed the specificity of the antibody.

(B) Western blot analysis of an immunoprecipitation experiment showing that RPTP69d does not diminish Robo3 phosphorylation in S2 cells.

(C–J) RPTP69d gain-of-function phenotype in sLNv neurons is independent of its phosphatase activity and intracellular domain. (C) Schematic showing the different RPTP mutant forms used in the experiments. (D) Wild-type animals showing the normal pattern of sLNv neurons. (E) Animals expressing RPTP69d full length. (F) RPTP69d mutant in the catalytic domain (DA3). (G) RPTP69d lacking the intracellular domain (ΔC). (H) RPTP69d lacking the extracellular domain (ΔN). (I) Only the extracellular domain of RPTP69d. (J) Quantification of the phenotypes observed upon expression of different RPTP mutant forms (data presented as mean ± SEM; ***$p < 0.001$, one-way ANOVA). A decrease in the length of the sLNv dorsal projection is observed with full-length RPTP69d, the catalytic mutant RPTP69dDA3, and RPTP69d-ΔC, while RPTP69d-ΔN and RPTP69dextra did not show defects. A.U., arbitrary units; ns, not significant. Scale bar, 20 μm.

RPTP69d Regulates Robo3 Independently of Its Phosphatase Activity

Next, we examined the molecular nature of the interaction between RPTP69d and Robo receptors. We focused on Robo3 because it showed the strongest loss-of-function phenotype in sLNv, and little is known about the molecular regulation of its activity. Work in the embryonic midline suggests that Robo1 activity is regulated by dephosphorylation of a conserved tyrosine. We started by asking whether Robo3 is tyrosine phosphorylated. A GFP-tagged Robo3 was expressed in *Drosophila* S2 cells, immunoprecipitated, and probed for tyrosine phosphorylation. We find that Robo3, which bears many tyrosine residues, is indeed tyrosine phosphorylated, as confirmed by λ-phosphatase treatment (Figure 5A). Next, we asked whether this phosphorylation is RPTP69d dependent. Surprisingly, RPTP69d co-expression did not reduce tyrosine phosphorylation of Robo3 (Figure 5B) even in the presence of Slit (Figure S5), suggesting that RPTP69d may not be a Robo3 phosphatase, and may regulate Robo3 activity by a different mechanism. To further examine this issue, we asked which domains of RPTP69d are important for its function in vivo. We expressed four mutant forms of RPTP69d in the sLNv: a phosphatase domain mutant (RPTP69d-DA3); a C-terminal deletion mutant (RPTP69d-ΔC), removing both phosphatase domains; an extracellular domain (N-terminal) deletion mutant (RPTP69d-ΔN) removing the Ig and fibronectin III (FNIII) domains, but maintaining the signal peptide plus juxtamembrane and transmembrane regions; and a construct including only the extracellular domain (RPTP69d-extra). We tested all these forms in the sLNv axonal repulsion assay. Both RPTP69d-DA3 and RPTP69d-ΔC mutants caused a significant decrease in sLNv axonal growth indistinguishable from the wild-type RPTP69d (Figures 5D–5G and 5J). Therefore, phosphatase activity is dispensable for RPTP69d function in axonal Robo3-dependent axonal repulsion. In contrast, the RPTP69d-ΔN and RPTP69d-extra mutant fails to decrease axonal length (Figures 5H–5J), indicating a requirement for the membrane-tethered extracellular domains of RPTP69d in regulating Robo3-dependent axonal growth in vivo.

Robo3 and RPTP69d Can Form Receptor Complexes

Whereas physical interactions between RPTPs, including RPTP69d, and their substrates are known to be very transient and difficult to detect by co-immunoprecipitation (Dascenco et al., 2015; Flint et al., 1997) we were able to readily co-immunoprecipitate Robo3 and RPTP69d from S2 cells (Figures 6A–6C). RPTP69d-ΔC and RPTP69d-ΔN were also able to bind Robo3, indicating that the interaction may require the transmembrane and/or the juxtamembrane domain of RPTP69d. Together, these data indicate that RPTP69d binds Robo3 but that binding and functional interactions do not require enzymatic activity of RPTP69d.

RPTP69d Increases Surface Presentation of Robo3

Since RPTP69d does not regulate Robo3 through its phosphatase activity, we decided to examine alternatives. One possibility is that RPTP69d directly binds to Slit and in this way increases

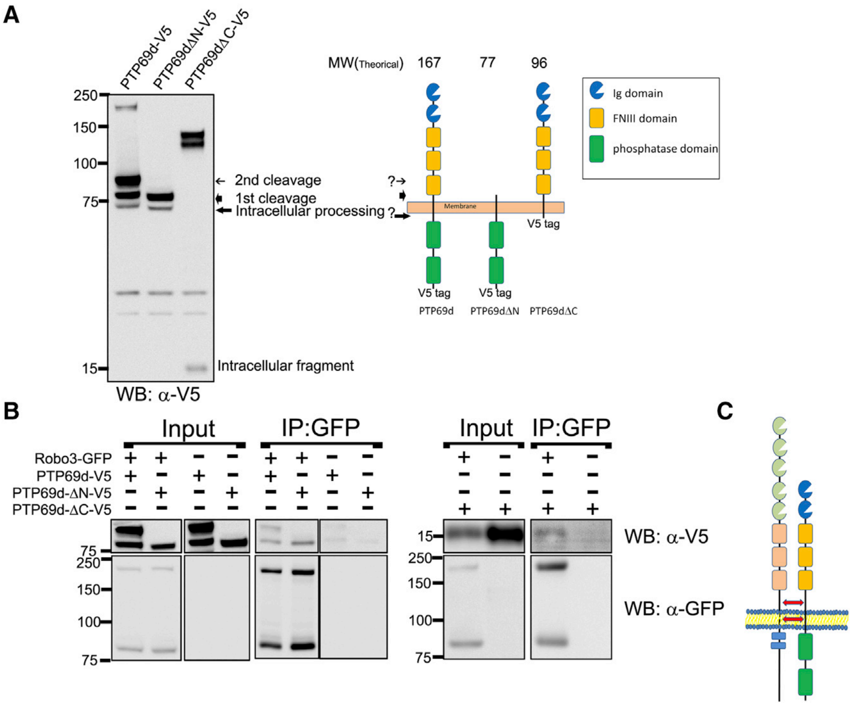

Figure 6. Robo3 Binds to RPTP69d through Its Transmembrane/Juxtamembrane Domain

(A) Western blot analysis of the RPTP constructs that were tested to immunoprecipitation with Robo3 (possible cleavage products are indicated by arrows). Schematic showing the domain constitution of the different RPTP69d constructs used in this experiment. Question marks indicate presumptive cleave sites.

(B) Co-immunoprecipitation showing that Robo3 is found in a protein complex with RPTP69d. Robo3 binds also to RPTP69d-ΔN and RPTP69d-ΔC. Note that in the case of the C-terminal deletion, a small fragment predicted to be the transmembrane domain plus some residues of the extracellular region (approximately 100) and 10 residues of the intracellular region have to co-precipitate with Robo3 to give rise to the observed 15 kDa.

(C) Schematic representing the proposed intracellular/juxtamembrane interaction between Robo3 and RPTP69d.

See also Figure S5.

Slit at the membrane, where it can bind to Robo and activate the pathway. However, although Robo3 co-immunoprecipitated RPTP69d and Slit, RPTP69d did not bind Slit in the absence of Robo3 (Figures 7A and 7B). Another possibility is that RPTP69d regulates Robo3 shedding, a recently proposed mechanism of Robo signaling activation (Coleman et al., 2010). Although we observed shedding of Robo3 in cell extracts, the level of processing was not significantly changed in the presence of RPTP69d (Figures S6A–S6E). We attempted to create an uncleavable form of Robo3, replacing the FNIII domains in the extracellular domain by the first FNIII domains of Frazzled, the same modification used for generating an uncleavable Robo (Coleman et al., 2010). However, we found no difference in the cleavage pattern between mutant (Robo3-[fraFN]) and wild-type Robo3. Interestingly, however, this form failed to co-immunoprecipitate RPTP69d (Figure S6F).

Finally, we considered the possibility that RPTP69d regulates Robo3 cell surface presentation. The molecules regulating Robo presentation outside the *Drosophila* VNC are largely unknown. To test whether RPTP69d might be such a regulator, we measured the amount of Robo3 on the axonal surface of primary *Drosophila* neurons (Figures 7C–7G). We expressed the same Robo3-GFP used in the in vivo experiments (intracellular, C-terminal tag) alone or in combination with wild-type RPTP69d, RPTP69d-ΔC, or RPTP69d-ΔN in primary *Drosophila* embryonic neurons. We used antibodies against the extracellular domain of Robo3 in the absence of detergents to measure Robo3 levels at the axonal surface, and used GFP fluorescence to determine the total levels of Robo3-GFP. We confirmed that intracellular antibodies do not stain in this condition (Figures S6G–S6H′). Next, we calculated the ratio of surface-to-total Robo3 alone or in the presence of various forms of RPTP69d at the growth cone. We find that wild-type RPTP69d and RPTP69d-ΔC, but not RPTP69d-ΔN, result in a significant increase of the extracellular Robo3 signal, indicating an increased abundance in Robo3 on the axonal surface (Figures 7C–7G; insets show extracellular Robo3 alone). This suggests that RPTP69d increases Robo3 levels on the cell surface, consistent with a role in enhancing Robo function. To provide direct biochemical evidence for this interaction, we used a cell surface biotinylation assay to measure the amount of Robo on the cell surface (Figures 7H and 7I). We observe that indeed co-expression of Robo3 with RPTP69d increases Robo3 levels on the cell surface (immunoprecipitate/input ratio). Since the mutant form of Robo3 (Robo3-[fraFN]) did not bind RPTP69d, we used it as negative control in these assays. We find that RPTP69d has no effect on the cell surface fraction of Robo3-[fraFN] (Figures 7H and 7I), showing that RPTP69d binding is essential for increased cell surface levels. Finally, we measured the amount of Robo3 in the membrane of culture cells using an antibody feeding assay (Figures 7J–7L). Cells were transfected with Robo3-GFP alone or with RPTP69d-V5 and treated with anti-Robo-extracellular and anti-GFP to compare the levels of Robo3 on the surface with total levels of Robo3 (see Experimental Procedures for details). We quantified the levels of Robo on the cell surface when expressed alone or together with RPTP69d, and observed a significant increase at the membrane in the presence of RPTP69d (Figures S6I–S6P′, for viewing separate channels). Therefore, in three independent assays we find that the presence of RPTP69d significantly increases levels of Robo3 receptors at the cell surface, including that of axonal growth cones. Together, our data indicate that RPTP69d enhances Robo(s) function by binding to it (Figure 6) and increasing its cell surface availability (Figure 7).

DISCUSSION

This work reveals a previously unknown function of MB in the *Drosophila* brain whereby neuropile-neuropile interactions guide axonal growth. Interestingly, a single subtype of neurons, the MB

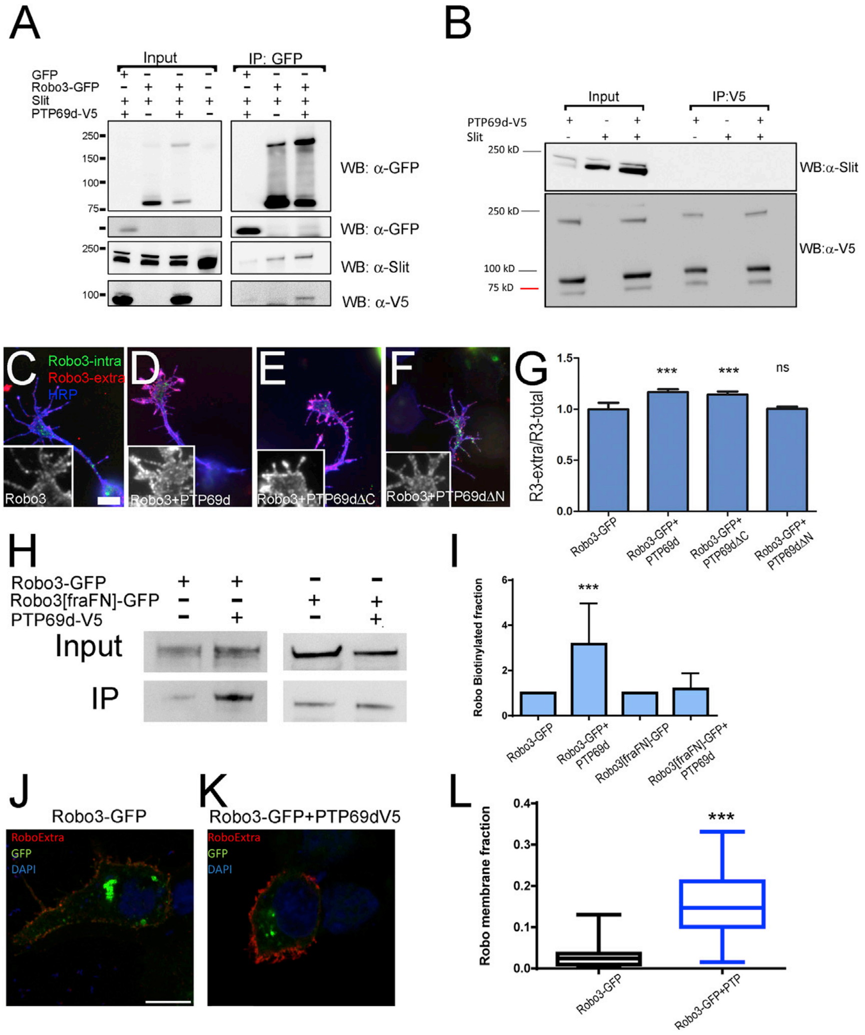

Figure 7. RPTP69d Increases Surface Presentation of Robo3

(A) Co-immunoprecipitation experiments showing that Robo3 is able to co-immunoprecipitate Slit and RPTP69d from S2 cells.

(B) Immunoprecipitation experiment of RPTP69d in the presence of Slit. Note that RPTP69d is not able to bind Slit.

(C–G) Primary cultures of embryonic neurons expressing *UAS-Robo3-GFP* (intracellular tag) in combination with different RPTP69d forms driven by *elav-Gal4* transactivator. Immunofluorescence using an extracellular Robo3 antibody (red) and GFP signal (green) detecting Robo3-GFP in non-permeabilized neurons was used to determine the extracellular/intracellular ratio. (C) Neurons expressing only Robo3-GFP. (D) Neurons expressing Robo3-GFP together with RPTP69d full length. (E) Neurons expressing Robo3-GFP and RPTP69d-ΔC. (F) Neurons expressing Robo3-GFP plus RPTP69d-ΔN. Insets show Robo3-extracellular staining alone. (G) Quantification of the ratio Robo3 extracellular/total for each condition (data are presented as mean ± SEM; ***p < 0.001, Mann-Whitney test). RPTP69d full length and ΔC, but not RPTP69d-ΔN, increase membrane Robo3 levels. Scale bar, 5 μm.

(H and I) Biotinylation assay (H) shows that more Robo3 is on the membrane in the presence of RPTP69d. (I) Quantification of the Robo3 biotinylated fraction in each condition (data are presented as mean ± SD; ***p < 0.001, two-tailed t test).

(J–L) Surface detection of Robo3 in cell culture. HEK293T cells expressing Robo3-GFP (intracellular tag) alone (J) or in combination with RPTP69d (K) were stained under non-permeabilizing conditions with Robo3 extracellular antibody (red). (L) Quantification of the Robo3 membrane fraction (data are presented as mean ± SD; ***p < 0.001, two-tailed t test). Scale bar, 10 μm.

See also Figure S6.

Kenyon cells, acts as a major source of Slit in the post-embryonic central brain. The reach of the axonal and dendritic arbors of these neurons covers significant areas of both developing brain hemispheres. This allows Kenyon cells to exert profound effects on neighboring neuronal circuits. Consistent with this, we observe significant defects in various brain fibers and neuropils close to the MB. We also uncover a Slit/Robo signaling mechanism that relies on an RPTP, specifically RPTP69d, as a co-receptor necessary to stabilize Robo cell surface levels and enhance its signaling activity. Interestingly, RPTP69d binds to Robo3 but does not require its phosphatase domain to mediate its positive effects on Robo3 function as a repulsive receptor.

The Mushroom Body Is a Source of Axonal Cues

In the embryo, midline glia expresses not only Slit but also the attractive cue Netrin (Brankatschk and Dickson, 2006; Kennedy et al., 1994; Kolodziej et al., 1996; Serafini et al., 1994). Thus, the midline constitutes an organizing center for VNC connectivity. The MB also expresses Netrin, and we find that its receptor Frazzled is required for growth of the sLNv axons (Figures S6Q–S6U). This suggests that the MB acts as source of both attractive and repulsive factors. Previous work (Nicolas and Preat, 2005) showed defects in the MB and the central complex, a structure close to the MB, in *robo2* and *robo3* mutants. The fact that the MB is a major source of Slit in the central brain explains these observations. We find that the MB itself does not express any of the Robo proteins at the larval stage, and previously it was shown that Robo2 and Robo3 are absent from MB in pupae (Nicolas and Preat, 2005), yet it has been reported to show defects in *robo2/3* mutants (Nicolas and Preat, 2005). This suggests a feedback mechanism between the MB and surrounding neuropils downstream of Slit/Robo signaling. MB neuroblasts are the first to be activated when the larval brain begins its expansion to form the adult brain (Ito and Hotta, 1992; Prokop and Technau, 1994), and the MB grows during development as the brain incorporates more neurons and circuit. The finding that the MB is a major source of Slit in the central brain may have implications for the interpretation of studies using MB structural or developmental mutants to study behavior.

Differential Regulation of Brain Wiring

Our observations show a different behavior of brain axons in response to Slit/Robo signaling. In the VNC, axons use Slit to

choose whether to cross or not to cross the midline and to select the appropriate longitudinal pathway among the three available tracts. In the central brain, sLNv axons do not change their trajectory in response to the increase or decrease of Slit; instead they change the length of the projection which is perpendicular to the Slit source (the calyx). This may be the consequence of the interaction between several guidance cues and cell-cell adhesive interactions. Furthermore, although most DCN axons are repelled from MB upon Robo2 ectopic expression, some of them appear to be attracted. This can be the results of Slit itself acting as an attractive cue for these axons or in combination with other guidance cues. Emergent properties of combinations of guidance cues have been already described in other systems. For instance, in the guidance of thalamocortical axons (TCA) Slit enables Netrin to attract TCA axons (Leyva-Diaz et al., 2014). For full understanding of the complex wiring of the brain it will eventually be necessary to characterize the expression patterns of guidance cues and their receptors, and to study their effects in combinations using live-imaging techniques.

A Distinct Mechanism of Robo Regulation in the Brain

During axon guidance at the midline, temporal regulation of the surface presentation of Robo receptors seems to be an important aspect. In *Drosophila* embryonic commissural axons, this is achieved by the Comm protein. However, in mammals, and even other insects, there are no Comm orthologs, indicating that Robo regulation by Comm is a non-conserved mechanism. Thus, other mechanisms of regulating Robo activity and surface availability remain to be discovered. Here we report that RPTP69d can form a complex with Robo3, thereby increasing its cell surface availability. These data suggest that direct regulation of Robo surface availability by RPTPs during axon growth represents an alternative mechanism to Comm-mediated regulation of Robo activity. RPTPs are conserved through evolution and several studies from invertebrate and vertebrate models have provided evidence of important roles of RPTPs in regulating axon guidance (Gatto et al., 2013; Stepanek et al., 2005), although the respective molecular pathways remain to be characterized. We speculate that the role of RPTPs in the Slit/Robo pathway might be an important mechanism for regulating Slit/Robo signaling in most systems.

It is interesting to note that RPTP69d positively regulates Robo receptor function independent of its enzymatic activity, in contrast to its function in guiding peripheral retinal axons (Garrity et al., 1999) and its phosphatase-dependent ability to regulate signaling of other co-receptors (Dascenco et al., 2015). *Drosophila* Lar has also been suggested to function independently of enzymatic activity, but the mechanism remains unknown (Hofmeyer and Treisman, 2009). This may point to RPTPs as dual-activity molecules acting as phosphatases for some guidance receptors, and as co-receptors or chaperones for others. In vertebrates, a major control mechanism for Slit/Robo-dependent repulsion in the spinal cord and the brain appears to be via the divergent ROBO3 receptor, which is mutated in horizontal gaze palsy with progressive scoliosis (Jen et al., 2004). ROBO3 antagonizes the repulsive effects of ROBO1 and ROBO2 (Marillat et al., 2004; Sabatier et al., 2004), yet the molecular underpinnings of this antagonism are unclear. It has also been suggested that signaling and surface presentation of ROBO1/2 can be promoted by co-overexpression of RabGDI (Philipp et al., 2012), but in the absence of mutant analysis it is unclear whether, how, and under what conditions this might occur in vivo. It would therefore be interesting to examine whether ROBO3 and or RabGDI—perhaps through interactions with RPTPs—regulates surface presentation of ROBO1/2.

EXPERIMENTAL PROCEDURES

Fly Culture

Flies were cultures on standard fly food. All experiments were performed under temperature-controlled conditions at 25°C or 28°C (RNAi experiments). Flip-out clones were generated by a 1-hr heat shock at 37°C for 2 days during pupal development.

Cloning

Standard molecular biology techniques were used to make the different RPTP69d constructs and the Robo3-[fraFN]-GFP construct.

Antibody Staining of *Drosophila* Brains

For adult and larval brain staining, animals were dissected in PBS, fixed, and stained using standard procedures. The following antibodies were obtained from the Developmental Studies Hybridoma Bank: mouse anti-Slit (1:20), mouse anti-Robo (1:50), mouse anti-Robo3 extracellular (1:50), mouse anti-PDH (1:50), mouse anti-Fasciclin2 (1:50), rat anti N-cadherin (1:10). Other antibodies used were: rabbit anti-NetA and rabbit anti-Robo2 (gifts from Barry Dickson; 1:1,000 and 1:500, respectively), mouse anti-GFP 3E6 (Invitrogen, catalog #A11120, 1:250), rabbit anti-GFP (Invitrogen, #A11122, 1:500) rabbit anti-Robo2 (1:1,000), rabbit anti-DsRed (Clontech, #632496; 1:500), and anti-HRP (Cy5-conjugated, Jackson ImmunoResearch, 1:50). Secondary antibodies conjugated with Alexa 488, Alexa 555, and Alexa 647 were obtained from Invitrogen and used at 1:500.

Cell Culture

For analysis of Robo3 localization, *Drosophila* embryonic primary neurons were used. HEK293T cells were used for antibody feeding experiments.

Immunoprecipitation

S2 cells were cultured in 6-well plates at 25°C in Sf900II medium. Electroporation was carried out using the Amaxa V kit (Lonza). For Slit treatment, Slit was obtained from the supernatant of S2 stable-expressing cells and was added 3 days after transfection. Six hours later the cells were extracted and the pellet frozen.

Cells were lysed using 400 μL of RIPA buffer and fresh added protease inhibitors cocktail 100× (Amresco). NaF and Na_3VO_4 phosphatase inhibitors were added if detection of tyrosine phosphorylation was required. Immunoprecipitation was carried out using anti-GFP conjugated beads (Chromotek).

Imaging

Imaging was performed using Leica SP5 and SP6 confocal microscopes (Wetzlar). Images were processed using ImageJ software (NIH). Figures were prepared using Adobe Photoshop (Adobe).

Immunoprecipitation

S2 cells were cultured in 6-well plates at 25°C in Sf900II medium. Transfections were performed with indicated constructs. Cells were lysed using RIPA buffer including NaF and Na_3VO_4 phosphatase inhibitors. anti-GFP conjugated beads (Chromotek) were used for immunoprecipitation.

Western Blotting

SDS-PAGE was performed using 4%–12% gradient polyacrylamide gels and then transferred to nitrocellulose membranes, according to standard protocols.

Statistics

Statistical analysis was performed using Prism software (GraphPad). For sLNv measurements, results are presented as a.u. representing the fraction

between the lengths of the dorsal projections divided by the distance between cell bodies in the two brain hemispheres. A two-tailed t test was used for analysis of two-group comparisons and ANOVA was used for multiple comparisons. For primary neuronal culture experiments the Mann-Whitney test was employed. For analysis of Robo phenotypes in DCN neurons, Fisher's exact test was performed.

Additional protocols and details are described in Supplemental Experimental Procedures.

SUPPLEMENTAL INFORMATION

Supplemental Information includes Supplemental Experimental Procedures and six figures and can be found with this article online at http://dx.doi.org/10.1016/j.devcel.2016.09.028.

AUTHOR CONTRIBUTIONS

Conceptualization, C.O., A.S., N.S.-S., and B.A.H.; Investigation, C.O., A.S., N.M., A.R., and N.S.-S.; Resources, N.D.G., A.C., M.-L.E., J.S., D.D., and D.S.; Software, R.K.E.; Writing – Original Manuscript, C.O. and B.A.H.; Writing – Review & Editing, C.O., A.S., B.A.H., D.S., and N.M.; Supervision, B.A.H.; Funding Acquisition, B.A.H., N.S.-S., D.S., and C.O.

ACKNOWLEDGMENTS

This work was supported by VIB (B.A.H., D.S.), the WiBrain Interuniversity Attraction Pole (BELSPO IUAP) network (B.A.H., D.S.), Fonds Wetenschappelijke Onderzoek (FWO) grants G.0543.08, G.0680.10, G.0681.10, and G.0503.12 (B.A.H.), ICM-P09015F (J.S.), FONDECYT 11150610 (C.O.), the Wellcome Trust (N.S.-S., grant to Manchester fly facility 087742/Z/08/Z), the Biotechnology and Biological Sciences Research Council (N.S.-S., grants BB/M007456/1 and BB/I002448/1), the Universities of Liverpool and Manchester (N.S.-S.), a postdoctoral fellowship from Becas Chile to C.O., University of Leuven postdoctoral fellowship (PDM) to A.S., and FWO postdoctoral fellowships to N.M and D.D. We are grateful to Somersault18:24 for help with schematics and to members of the Hassan laboratory for discussions. B.A.H. is an Allen Distinguished Investigator and an Einstein Visiting Fellow.

Received: March 11, 2015
Revised: March 29, 2016
Accepted: August 25, 2016
Published: October 24, 2016

REFERENCES

Bashaw, G.J., Kidd, T., Murray, D., Pawson, T., and Goodman, C.S. (2000). Repulsive axon guidance: Abelson and Enabled play opposing roles downstream of the roundabout receptor. Cell *101*, 703–715.

Brankatschk, M., and Dickson, B.J. (2006). Netrins guide *Drosophila* commissural axons at short range. Nat. Neurosci. *9*, 188–194.

Chagnon, M.J., Uetani, N., and Tremblay, M.L. (2004). Functional significance of the LAR receptor protein tyrosine phosphatase family in development and diseases. Biochem. Cell Biol. *82*, 664–675.

Chedotal, A. (2011). Further tales of the midline. Curr. Opin. Neurobiol. *21*, 68–75.

Chilton, J.K. (2006). Molecular mechanisms of axon guidance. Dev. Biol. *292*, 13–24.

Clandinin, T.R., Lee, C.H., Herman, T., Lee, R.C., Yang, A.Y., Ovasapyan, S., and Zipursky, S.L. (2001). *Drosophila* LAR regulates R1-R6 and R7 target specificity in the visual system. Neuron *32*, 237–248.

Coleman, H.A., Labrador, J.P., Chance, R.K., and Bashaw, G.J. (2010). The Adam family metalloprotease Kuzbanian regulates the cleavage of the roundabout receptor to control axon repulsion at the midline,. Development *137*, 2417–2426.

Dascenco, D., Erfurth, M.L., Izadifar, A., Song, M., Sachse, S., Bortnick, R., Urwyler, O., Petrovic, M., Ayaz, D., He, H., et al. (2015). Slit and receptor tyrosine phosphatase 69D confer spatial specificity to axon branching via Dscam1. Cell *162*, 1140–1154.

Dickson, B.J., and Gilestro, G.F. (2006). Regulation of commissural axon pathfinding by slit and its Robo receptors. Annu. Rev. Cell Dev. Biol. *22*, 651–675.

Flint, A.J., Tiganis, T., Barford, D., and Tonks, N.K. (1997). Development of "substrate-trapping" mutants to identify physiological substrates of protein tyrosine phosphatases. Proc. Natl. Acad. Sci. USA *94*, 1680–1685.

Garrity, P.A., Lee, C.H., Salecker, I., Robertson, H.C., Desai, C.J., Zinn, K., and Zipursky, S.L. (1999). Retinal axon target selection in *Drosophila* is regulated by a receptor protein tyrosine phosphatase. Neuron *22*, 707–717.

Gatto, G., Dudanova, I., Suetterlin, P., Davies, A.M., Drescher, U., Bixby, J.L., and Klein, R. (2013). Protein tyrosine phosphatase receptor type O inhibits trigeminal axon growth and branching by repressing TrkB and Ret signaling. J. Neurosci. *33*, 5399–5410.

Godenschwege, T.A., Simpson, J.H., Shan, X., Bashaw, G.J., Goodman, C.S., and Murphey, R.K. (2002). Ectopic expression in the giant fiber system of *Drosophila* reveals distinct roles for roundabout (Robo), Robo2, and Robo3 in dendritic guidance and synaptic connectivity. J. Neurosci. *22*, 3117–3129.

Heisenberg, M., Borst, A., Wagner, S., and Byers, D. (1985). *Drosophila* mushroom body mutants are deficient in olfactory learning. J. Neurogenet. *2*, 1–30.

Helfrich-Forster, C., Shafer, O.T., Wulbeck, C., Grieshaber, E., Rieger, D., and Taghert, P. (2007). Development and morphology of the clock-gene-expressing lateral neurons of *Drosophila melanogaster*. J. Comp. Neurol. *500*, 47–70.

Hofmeyer, K., and Treisman, J.E. (2009). The receptor protein tyrosine phosphatase LAR promotes R7 photoreceptor axon targeting by a phosphatase-independent signaling mechanism,. Proc. Natl. Acad. Sci. USA *106*, 19399–19404.

Ito, K., and Hotta, Y. (1992). Proliferation pattern of postembryonic neuroblasts in the brain of *Drosophila melanogaster*. Dev. Biol. *149*, 134–148.

Jen, J.C., Chan, W.M., Bosley, T.M., Wan, J., Carr, J.R., Rub, U., Shattuck, D., Salamon, G., Kudo, L.C., Ou, J., et al. (2004). Mutations in a human ROBO gene disrupt hindbrain axon pathway crossing and morphogenesis. Science *304*, 1509–1513.

Jhaveri, D., Saharan, S., Sen, A., and Rodrigues, V. (2004). Positioning sensory terminals in the olfactory lobe of *Drosophila* by Robo signaling. Development *131*, 1903–1912.

Katsuki, T., Ailani, D., Hiramoto, M., and Hiromi, Y. (2009). Intra-axonal patterning: intrinsic compartmentalization of the axonal membrane in *Drosophila* neurons. Neuron *64*, 188–199.

Keleman, K., Rajagopalan, S., Cleppien, D., Teis, D., Paiha, K., Huber, L.A., Technau, G.M., and Dickson, B.J. (2002). Comm sorts robo to control axon guidance at the *Drosophila* midline. Cell *110*, 415–427.

Keleman, K., Ribeiro, C., and Dickson, B.J. (2005). Comm function in commissural axon guidance: cell-autonomous sorting of Robo in vivo. Nat. Neurosci. *8*, 156–163.

Kennedy, T.E., Serafini, T., de la Torre, J.R., and Tessier-Lavigne, M. (1994). Netrins are diffusible chemotropic factors for commissural axons in the embryonic spinal cord. Cell *78*, 425–435.

Kolodziej, P.A., Timpe, L.C., Mitchell, K.J., Fried, S.R., Goodman, C.S., Jan, L.Y., and Jan, Y.N. (1996). Frazzled encodes a *Drosophila* member of the DCC immunoglobulin subfamily and is required for CNS and motor axon guidance. Cell *87*, 197–204.

Krashes, M.J., Keene, A.C., Leung, B., Armstrong, J.D., and Waddell, S. (2007). Sequential use of mushroom body neuron subsets during *Drosophila* odor memory processing. Neuron *53*, 103–115.

Kraut, R., and Zinn, K. (2004). Roundabout 2 regulates migration of sensory neurons by signaling in *trans*. Curr. Biol. *14*, 1319–1329.

Langen, M., Koch, M., Yan, J., De Geest, N., Erfurth, M.L., Pfeiffer, B.D., Schmucker, D., Moreau, Y., and Hassan, B.A. (2013). Mutual inhibition among postmitotic neurons regulates robustness of brain wiring in Drosophila. Elife *2*, e00337.

Leyva-Diaz, E., del Toro, D., Menal, M.J., Cambray, S., Susin, R., Tessier-Lavigne, M., Klein, R., Egea, J., and Lopez-Bendito, G. (2014). FLRT3 is a

Robo1-interacting protein that determines Netrin-1 attraction in developing axons. Curr. Biol. *24*, 494–508.

Lowery, L.A., and Van Vactor, D. (2009). The trip of the tip: understanding the growth cone machinery. Nat. Rev. Mol. Cell Biol. *10*, 332–343.

Marillat, V., Sabatier, C., Failli, V., Matsunaga, E., Sotelo, C., Tessier-Lavigne, M., and Chedotal, A. (2004). The slit receptor Rig-1/Robo3 controls midline crossing by hindbrain precerebellar neurons and axons,. Neuron *43*, 69–79.

Nicolas, E., and Preat, T. (2005). *Drosophila* central brain formation requires Robo proteins. Dev. Genes Evol. *215*, 530–536.

Pappu, K.S., Morey, M., Nern, A., Spitzweck, B., Dickson, B.J., and Zipursky, S.L. (2011). Robo-3-mediated repulsive interactions guide R8 axons during *Drosophila* visual system development. Proc. Natl. Acad. Sci. USA *108*, 7571–7576.

Philipp, M., Niederkofler, V., Debrunner, M., Alther, T., Kunz, B., and Stoeckli, E.T. (2012). RabGDI controls axonal midline crossing by regulating Robo1 surface expression. Neural Dev. *7*, 36.

Pitman, J.L., McGill, J.J., Keegan, K.P., and Allada, R. (2006). A dynamic role for the mushroom bodies in promoting sleep in *Drosophila*. Nature *441*, 753–756.

Prokop, A., and Technau, G.M. (1994). Normal function of the mushroom body defect gene of *Drosophila* is required for the regulation of the number and proliferation of neuroblasts. Dev. Biol. *161*, 321–337.

Rajagopalan, S., Nicolas, E., Vivancos, V., Berger, J., and Dickson, B.J. (2000). Crossing the midline: roles and regulation of Robo receptors. Neuron *28*, 767–777.

Sabatier, C., Plump, A.S., Le, M., Brose, K., Tamada, A., Murakami, F., Lee, E.Y., and Tessier-Lavigne, M. (2004). The divergent Robo family protein rig-1/Robo3 is a negative regulator of slit responsiveness required for midline crossing by commissural axons. Cell *117*, 157–169.

Serafini, T., Kennedy, T.E., Galko, M.J., Mirzayan, C., Jessell, T.M., and Tessier-Lavigne, M. (1994). The netrins define a family of axon outgrowth-promoting proteins homologous to *C. elegans* UNC-6. Cell *78*, 409–424.

Siu, R., Fladd, C., and Rotin, D. (2007). N-cadherin is an in vivo substrate for protein tyrosine phosphatase sigma (PTPsigma) and participates in PTPsigma-mediated inhibition of axon growth. Mol. Cell. Biol. *27*, 208–219.

Stepanek, L., Stoker, A.W., Stoeckli, E., and Bixby, J.L. (2005). Receptor tyrosine phosphatases guide vertebrate motor axons during development. J. Neurosci. *25*, 3813–3823.

Sun, Q., Bahri, S., Schmid, A., Chia, W., and Zinn, K. (2000). Receptor tyrosine phosphatases regulate axon guidance across the midline of the *Drosophila* embryo. Development *127*, 801–812.

Tayler, T.D., Robichaux, M.B., and Garrity, P.A. (2004). Compartmentalization of visual centers in the *Drosophila* brain requires Slit and Robo proteins. Development *131*, 5935–5945.

Tear, G., Harris, R., Sutaria, S., Kilomanski, K., Goodman, C.S., and Seeger, M.A. (1996). Commissureless controls growth cone guidance across the CNS midline in *Drosophila* and encodes a novel membrane protein. Neuron *16*, 501–514.

Trunova, S., Baek, B., and Giniger, E. (2011). Cdk5 regulates the size of an axon initial segment-like compartment in mushroom body neurons of the *Drosophila* central brain. J. Neurosci. *31*, 10451–10462.

Zschatzsch, M., Oliva, C., Langen, M., De Geest, N., Ozel, M.N., Williamson, W.R., Lemon, W.C., Soldano, A., Munck, S., Hiesinger, P.R., et al. (2014). Regulation of branching dynamics by axon-intrinsic asymmetries in Tyrosine Kinase Receptor signaling. Elife *3*, e01699.

Developmental Cell, Volume *39*

Supplemental Information

Regulation of *Drosophila* Brain Wiring by Neuropil Interactions via a Slit-Robo-RPTP Signaling Complex

Carlos Oliva, Alessia Soldano, Natalia Mora, Natalie De Geest, Annelies Claeys, Maria-Luise Erfurth, Jimena Sierralta, Ariane Ramaekers, Dan Dascenco, Radoslaw K. Ejsmont, Dietmar Schmucker, Natalia Sanchez-Soriano, and Bassem A. Hassan

Supplemental Inventory

Supplemental Figure S1, Related to Figure 1.

Supplemental Figure S2, Related to Figure 2.

Supplemental Figure S3, Related to Figure 3.

Supplemental Figure S4, Related to Figure 4.

Supplemental Figure S5, Related to Figure 6.

Supplemental Figure S6, Related to Figure 7.

Supplemental Figure legends

Supplemental Experimental Procedures

Supplemental References

Figure S1, related to Figure 1

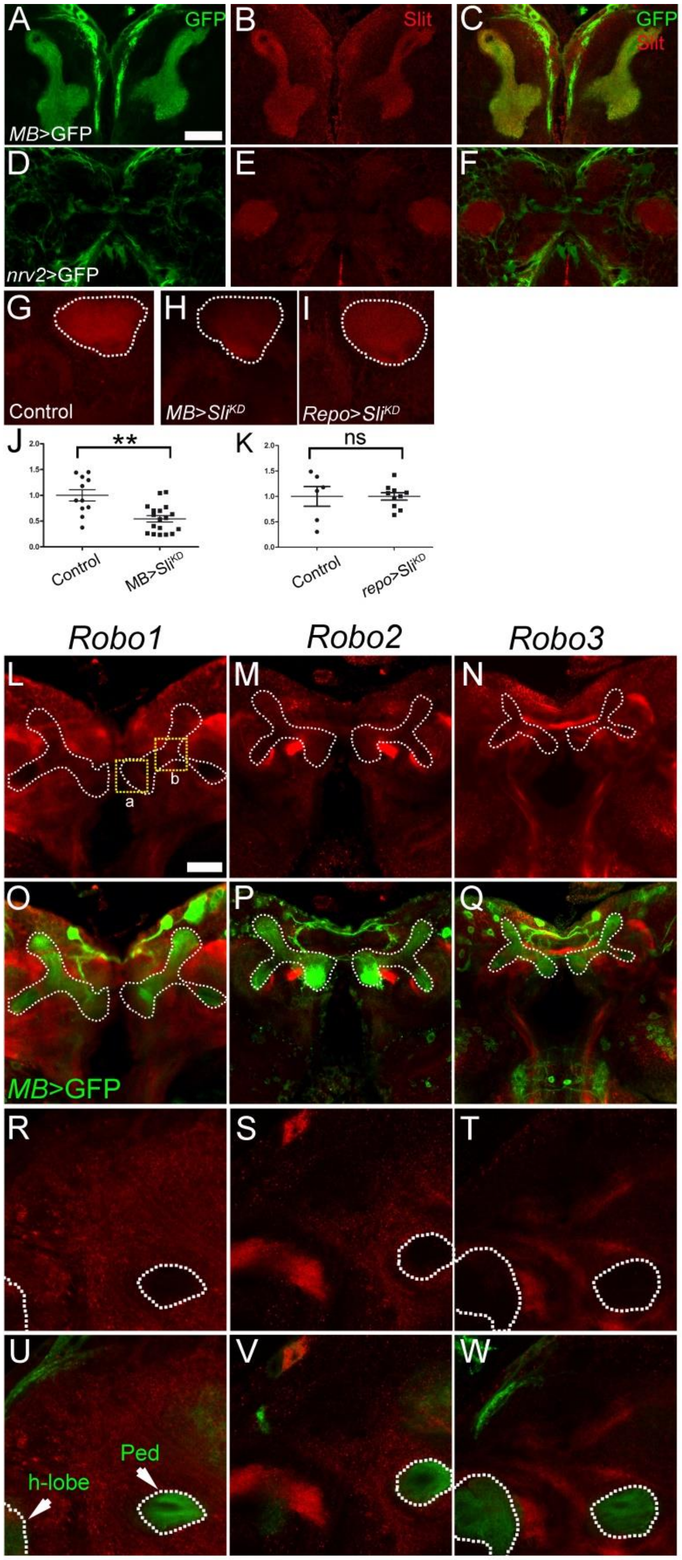

Figure S2, related to Figure 2

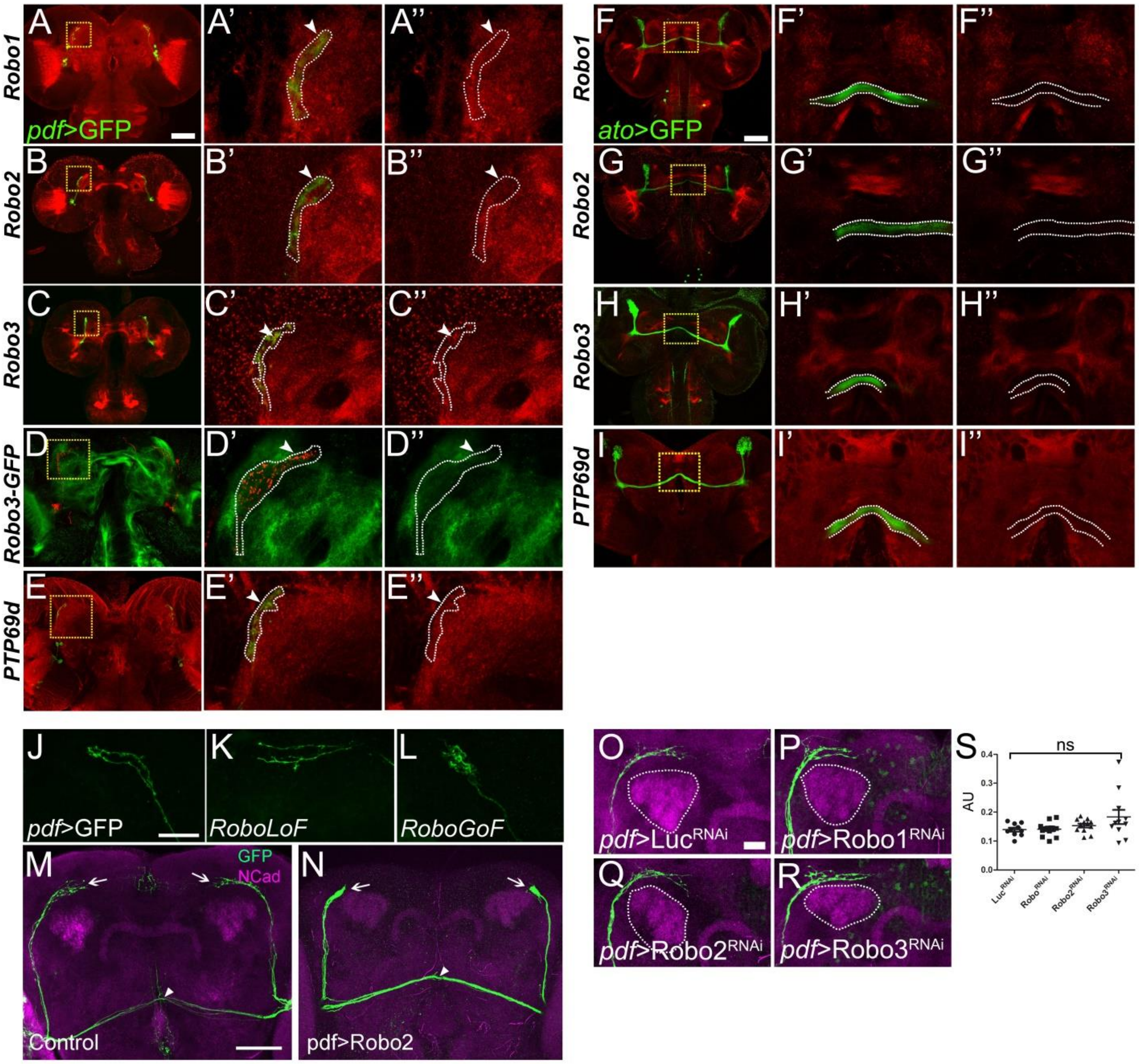

Figure S3, related to Figure 3

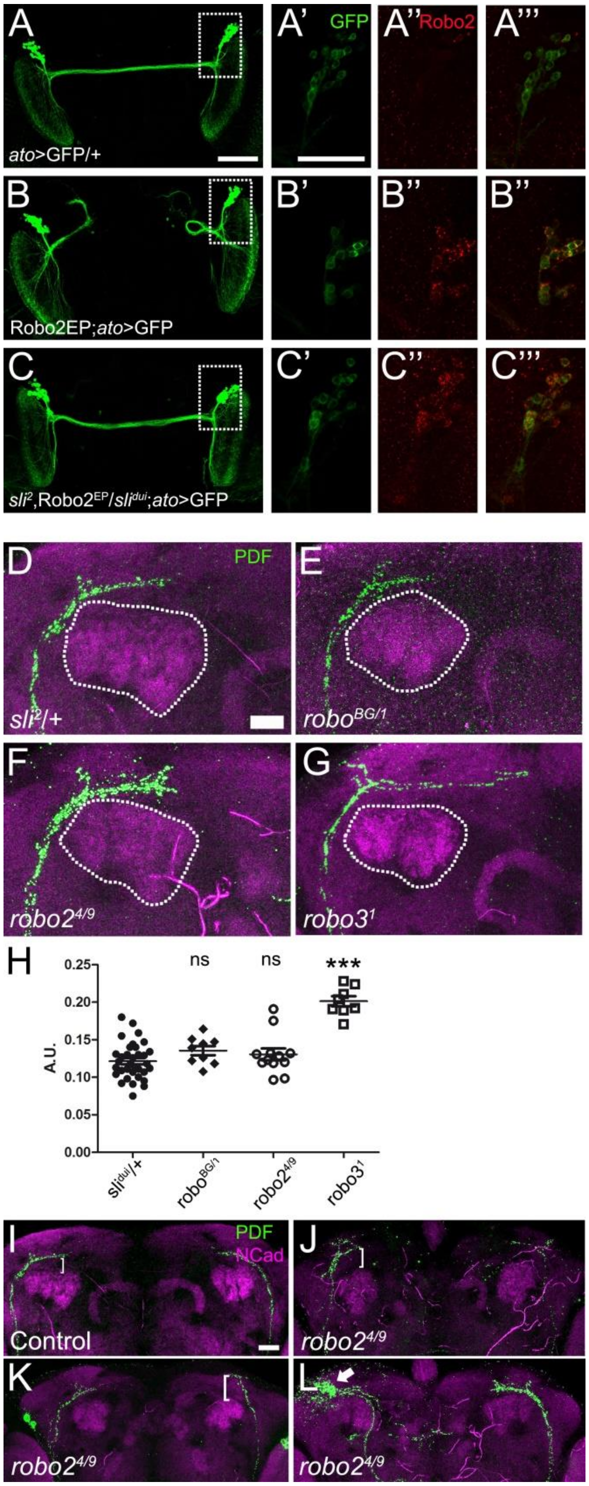

Figure S4, related to Figure 4

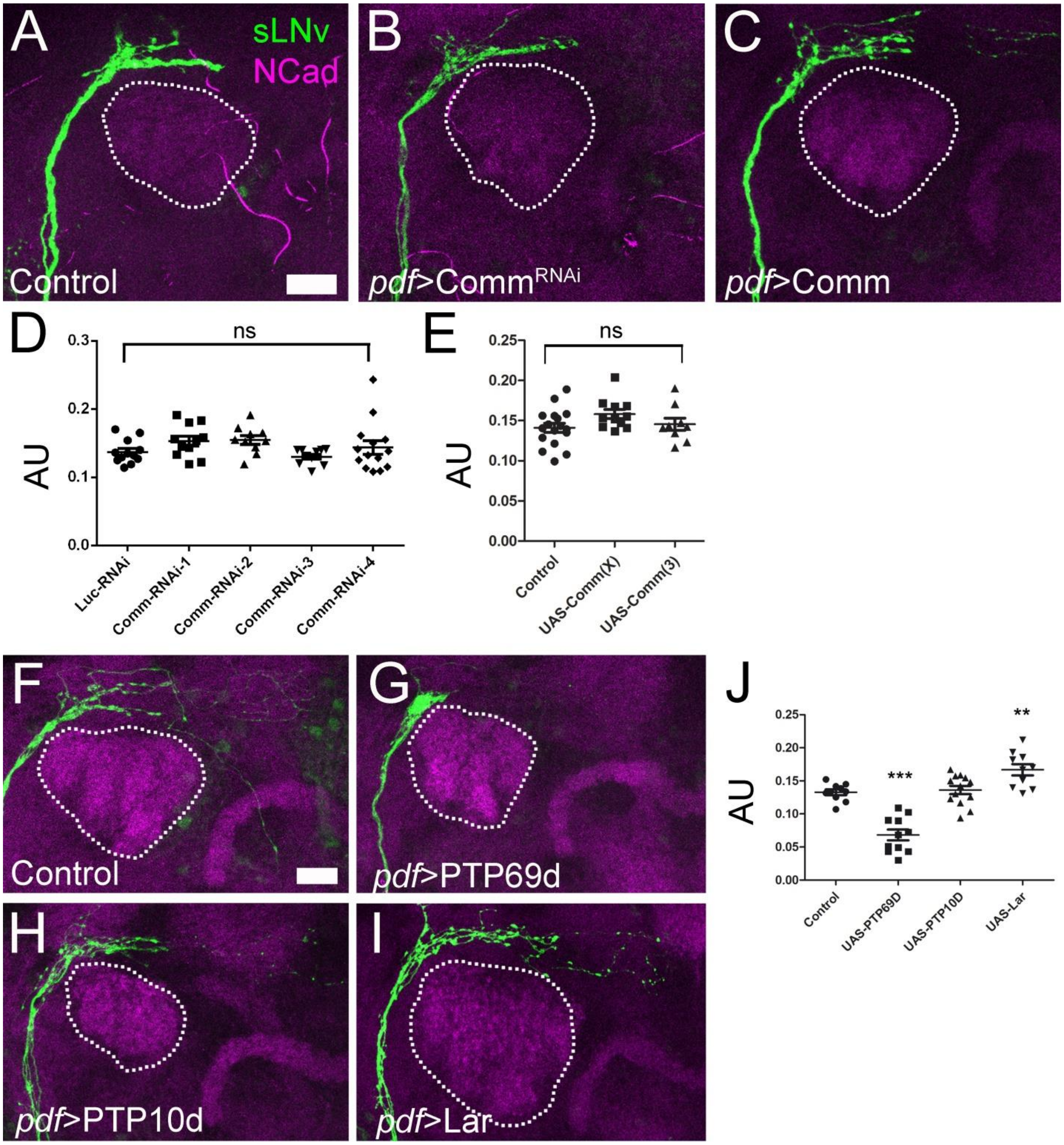

Figure S5, related to Figure 6

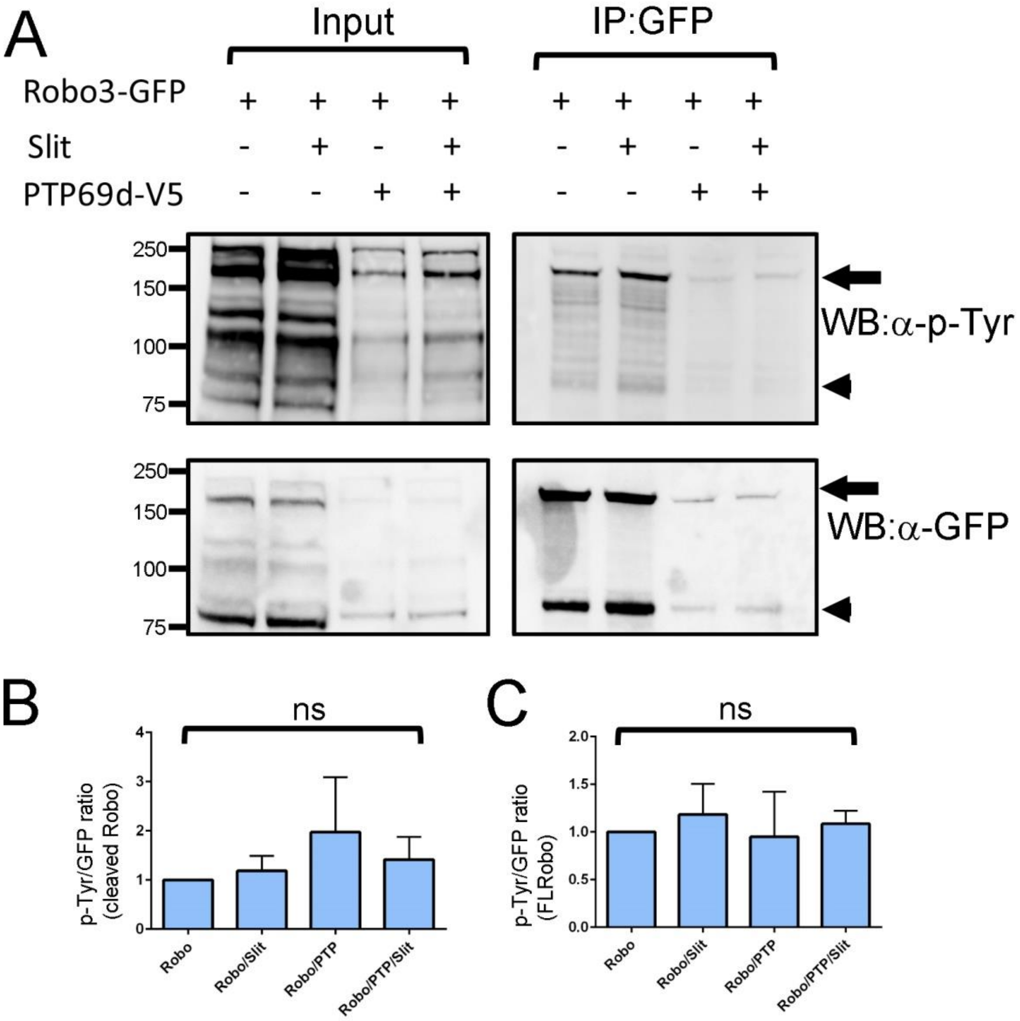

Figure S6 related to Figure 7

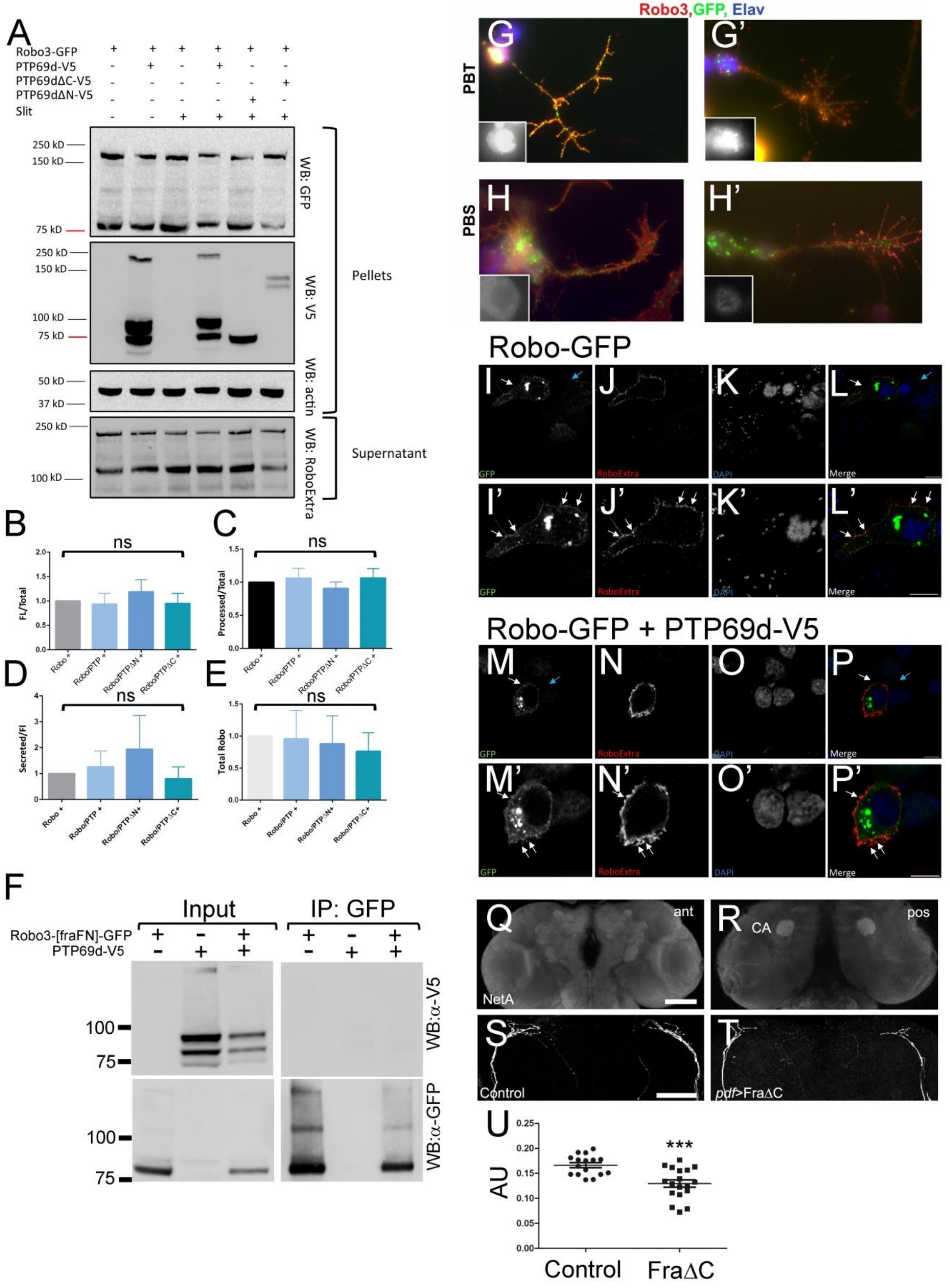

Supplemental Figure legends

FIGURE S1. Related to Figure 1. (A-K) Slit is expressed in the MB neuropile.

(A-F) Staining of larval brains with the indicated antibodies. (A-C) Slit (red) is expressed in the mushroom body neuropile, reported by GFP under the control of *OK107-Gal4* (green). (D-F) Absence of colocalization is observed with the wrapping glia marker *Nrv2-Gal4*, indicating that Slit is only in the neurons. (G, I) Staining of adult brains with Slit antibody in wild type (G), Slit knock-down (KD) in MB (H) and glial cells (I). The MB calyx is surrounded with a dashed line. (J, K) Quantification of Slit intensity (data presented as mean $\pm$ SEM, Mann-Whitney test, $^{**}p<0.01$).

(L-W) Robo expression patterns in the central brain. (L-Q) Staining of larval brains using the indicated antibodies, mushroom area is surrounded by dashed lines. (R-W) Close up views of the panels above (single sections) in regions indicated by yellow dashed squares. Note that Robo1, Robo2 and Robo3 show no significant expression in the MBs. Scale bar: 30 μm

FIGURE S2. Related to Figure 2. (A-E'') Expression pattern of Robos and RPTP69d in sLNv.

(A- C'') Analysis of the expression pattern of different Robo proteins (red) in s-LNv axons (green) in L3 stage. (A', A'', B', B'', C', C'') Close up views (single sections or projections of 2 to 3 optical slices of 0.8 um thickness) in regions indicated with yellow dashed squares, all Robo receptors show detectable expression (arrowheads), the area of s-LNv axons is surrounded by dashed lines. (D-D'') Expression analysis of Robo3 in L3 stage, using a GFP genomically-tagged line (RMCE-MIMIC) in the green channel, PDH antibody is used to highlight pdf neurons (red). (D', D'') Close up views acquired similar to above. (E, E'') Expression analysis of RPTP69d using a specific antibody (red), sLNv axons are depicted in green. (E', E'') Close up views acquired similar to above. RPTP69d is detected in sLNv axons (arrow in E' and E'').

Expression pattern of Robos and RPTP69d in DCN axons.

(F-H'') Expression pattern of different Robo proteins (red) in the DCN axons (green) in L3 stage. (F', F'', G', G'', H', H'') Close up views (single sections or projections of 2 to 3 optical slices of 0.8 um thickness) of regions indicated with yellow dashed squares. Note that none of the Robo receptors show significant expression above background levels. (I-I'') Expression analysis of RPTP69d protein (red) in DCN axons (green) in L3 stage. (I', I'') Close up views of the regions indicated by yellow dashed squares, acquired similar to above sections.

Single cell clones of sLNv phenotypes upon Robo signaling manipulation and phenotypes of lLNv commissure.

(J-L) Single sLNv axons (adult stage) labeled using *pdf-Gal4* in combination with a flip-out cassette (see Material and Methods section). (J) Control axon expressing GFP. (K) Axon expressing GFP and Robo2ΔC transgenes. (L) Axon expressing GFP and Robo2 transgenes. Notice that axons expressing Robo2ΔC overshoot their targets while those over-expressing Robo2 arrest before reaching its normal length. (M-N) Adult brains of animals bearing *pdf-Gal4* in combination with *UAS-GFP* reporter and stained with N-Cadherin antibody. (M) Control brains (arrowhead indicates lLNv commissure and arrows sLNv axon terminals). (N) Robo2 over-expression using *pdf-Gal4*, notice that sLNv terminals are shorter but lLNv commissures are unaffected. (O-S) Robo-RNAi phenotypes in the sLNv. (O-R) Staining of adult brains, bearing *UAS-Robo-RNAi* transgenes under the control of *pdf-Gal4*. Knocking down the different Robo proteins in sLNv separately do not elicit significant over-growth phenotypes, although a tendency is observed with the Robo3-RNAi. (S) Quantification of the observed phenotypes (data presented as mean $\pm$ SEM, One-way ANOVA). AU: arbitrary units.

Scale bars: (A-I''): 60 μm, (J,L): 20μm, (M,N): 60μm, (O,R): 20μm

FIGURE S3. Related to Figure 3.

(A-C''') Robo2 over-expression phenotypes in DCNs are Slit dependent. Adult brains bearing an *ato-Gal4* driver in combination with a *UAS-CD8-GFP* reporter. (A) Control DCN axons. (A'-A''') Close up view (single section or projection of 2 to 3 sections) of a region indicated by a dashed rectangle in (A), Robo2 staining is absent (red). (B) Brain over-expressing Robo2. (B'-B''') Close up view acquired similar to above of a region in (B), Robo2 staining is observed (red). (C) Brain over-expressing Robo2 in a *slit* mutant background. (C'-C''') Close up view acquired similar to above of a region in (C), Robo2 staining is present (red) but the commissure is not affected.

(D-H) sLNv phenotypes in *robo* mutants. (D-G) Adult brains bearing a *pdf-Gal4* driver in combination with a *UAS-CD8-GFP* reporter. (D) *sli/+* heterozigotes used as a control condition. (E) $robo^{1}/robo^{BG}$ transheterozigotes. (F) $robo2^{4}/robo2^{9}$ transheterozigotes. (G) $robo3^{1}$ homozigote mutants. (H) Quantification of the observed phenotypes (data presented as mean $\pm$ SEM One-way ANOVA, $^{***}p<0.001$).

(I-L) Early *robo2* mutant phenotypes of sLNv neurons. Adult brains of the indicated genotypes labeled with anti-PDH (green) and anti-N-Cadherin (magenta). (I) Control brain showing the sLNv wild type pattern. (J-K) Examples

of *robo2⁴/robo2⁹* showing axonal defects. (L) Example of *robo2⁴/robo2⁹* showing misplaced sLNv cell bodies (arrow).
AU: arbitrary units. Scale bars: (A, B, C): 80μm, (A'-A''', B'-B''', C'-C'''): 40μm, (D-L): 20μm.

FIGURE S4. Related to Figure 4.
(A-E) Commissureless does not act in sLNv axon growth. (A-C) Staining of adult brains bearing *UAS-Comm-RNAi* (B) or *UAS-Comm* (C) under the control of *pdf-Gal4*. Note that no effect is observed on the sLNv axon growth in any of the conditions. (D-E) Quantifications of the effects (data presented as mean ± SEM, One-way ANOVA).
(F-J) Effect of RPTPs over-expression in sLNv axon growth. (F-I) Staining of adult brains bearing transgenes *UAS-RPTP69d* (G), *UAS-RPTP10D* (H) and *UAS-LAR* (I). Note that RPTP9d over-expressing produces a decrease of the length of the sLNv axons. (J) Quantifications of the observed phenotypes (data presented as mean ± SEM, One-way ANOVA, **p<0.01, ***p<0.001). AU: arbitrary units.
Scale bars: 20 μm.

FIGURE S5. Related to Figure 6.
Slit does not affect Robo3 phosphorylation. (A) The indicated constructs were transfected in S2 cells. Robo3-GFP was IP'd from the cells extracts alone, or in the presence of PTP69d-V5 or/and Slit. IP'd fraction was tested for tyrosine phosphorylation and there were not significant changes among the conditions. (B) Quantification of tyrosine phosphorylation levels normalized by GFP signal measured for cleaved Robo3 (arrowheads) and (C) Robo full length (arrows), data presented as mean ± SD.

FIGURE S6. Related to Figure 7.
(A-E) Robo3 shedding is not affected by RPTP69d
(A) Western blot analysis of S2 cells transfected with the indicated constructs. Robo3 processing is not affected in the presence of PTP69d constructs and Slit. (B-E) Quantification of the observed changes in processing, data presented as mean ± SD.
(F) Robo3[fraFN] does not bind RPTP69d. Co-IP experiment showing that Robo3[fraFN]-GFP is not able to co-IP RPTP69d from S2 cells.
(G-H') Antibodies do not cross the cell membrane upon no-permeabilizing conditions. We tested if we were able to detect Elav protein when no detergent was used during the staining procedure. (G, G') Staining in primary *Drosophila* neurons using the indicated antibodies. GFP is detected directly without antibody. (H, H') The same staining performed above but in absence of detergent.
(I-P') Separated channels of experiments showed in Figure 7J-L. (I-L') Membrane localization of Robo3-GFP. (M-P') Membrane localization of Robo3-GFP in the presence of PTP69d.
(Q-U) NetrinA/Frazzled signaling regulates sLNv axon growth. (A-B) NetA expression in the L3 brain, (A) anterior view and (B) posterior view. Note high NetA expression in the calyx of the MB (B). (C, D) Adult brains stained with anti-PDH antibody, in (D) expression of Frazzled-ΔC (dominant negative) using *pdf-Gal4* driver. (E) Quantification of defects in the growth of sLNv axons (Data presented as mean ± SEM, two-tail T-test, ***p< 0,001). AU: arbitrary units. Scale bars: 60 μm.

Supplemental Experimental Procedures
Fly culture
Flies were cultures on standard fly food. All experiments were performed in temperature-controlled conditions at 25C or 28C (RNAi experiments). Flip-out clones were generated by a 1hr heat shock at 37C for two days during pupal development. The following fly strains were used:
(1) CS10
(2) *sli²*/CyO (Tayler et al., 2004)
(3) *sli^dui*/CyO (Tayler et al., 2004)
(4) *rptp69d¹* (Desai et al., 1996)
(5) *robo¹*/CyO
(6) *robo^BG*
(7) *robo2⁴*/CyO
(8) *robo2⁹*/CyO
(9) *robo3¹*/CyO

(10) *UAS-CD8-GFP* (BDSC)
(11) *UAS-Robo2-EGFP* (Katsuki et al., 2009)
(12) *UAS-Robo3-EGFP* (Katsuki et al., 2009)
(13) *UAS-Robo2-ΔC* (a gift from Greg Bashaw)
(14) *UAS-Robo1-ΔC* (a gift from Greg Bashaw)
(15) *UAS-Dicer2* (BDSC)
(16) *UAS-Robo-RNAi* (Tayler et al., 2004)
(17) *UAS-Robo2-RNAi* (Tayler et al., 2004)
(18) *UAS-Robo3-RNAi* (Tayler et al., 2004)
(19) *UAS-Luciferase-RNAi* (BDSC 31603)
(20) *UAS-RPTP69d-HA* (Dascenco et al., 2015)
(21) *UAS-RPTP10d*
(22) *UAS-LAR* (BDSC 9149)
(23) *UAS-RPTP69d-RNAi* (VDRC 104761)
(24) *UAS-Slit-RNAi* (BDSC 31468)
(25) *UAS-Slit-RNAi* (VDRC 38223)
(26) Df(2R)BSC482(*sli*)/CyO (BDSC)
(27) *sli*^{2}/CyO; *Slit-RNAi* (BDSC)
(28) Df(2R)BSC482(*sli*), Slit-RNAi (VDRC)/CyO
(29) *UAS-RPTP69d-ΔN-V5* (see methods)
(30) *UAS-RPTP69d-ΔC* (DGRC 109088)
(31) *UAS-RPTP69d-extracellular-V5* (see methods)
(32) *pdf-Gal4*
(33) *ato-Gal4*
(34) *elav-Gal4*
(35) *UAS-Dicer2; UAS-CD8-GFP; OK107-Gal4*
(36) *UAS-CD8-GFP; ato-Gal4, UAS-LacZ*
(37) *UAS-Dicer2; UAS-CD8-GFP/sli2; Slit-RNAi/+; OK107-Gal4*
(38) *UAS-Comm(X)* (a gift from Matthias Landgraf)
(39) *UAS-Comm(3)* (a gift from Barry Dickson)
(40) *UAS-Comm-RNAi-1* (BDSC 28381)
(41) *UAS-Comm-RNAi-2* (VDRC 5277GD)
(42) *UAS-Comm-RNAi-3* (VDRC 5278GD)
(43) *UAS-Comm-RNAi-4* (VDRC 110488KK)
(44) *UAS-Frazzled-ΔC* (a gift from Greg Bashaw)
(45) *Robo3MIMIC-GFP* (BDSC 60286)
(46) *hsFlp; UAS <CD2,Y>GFP*
(47) *GMR31A10-Gal4* (Janelia Farm)

Flybase gene references
slit: FBgn0264089; robo1: FBgn0005631; robo2: FBgn0002543; robo3: FBgn0002543; comm: FBgn0010105; Abelson: FBgn0000017 ; Ptp69D: FBgn0014007; Lar: FBgn0000464; Ptp10D: FBgn0004370; NetA: FBgn0015773; frazzled: FBgn0011592.

Cloning
cDNA for HA-tagged RPTP69D in pUAS-attB was obtained from DGRC (UFO0693). For the V5-tagged versions the full ORF except the stop-codon was PCR-amplified and TOPO cloned in frame with the V5 tag of the vector pIB-V5/HIS-TOPO (Life-Technologies).

<u>RPTP69dΔC:</u>
The N-terminal region of PTP69d (residues 1-834) including the transmembrane domain (residues 806-823) including a V5 tag was amplified by PCR and cloned into pUAST-attB vector.

<u>RPTP69dΔN:</u>
The signal peptide (residues 1-29) and the C-terminal region (residues 797-1462) including the transmembrane domain including a V5 tag were PCR amplified and fused by overlapping PCR. Then, digested with EagI-Acc65I

enzymes and ligated into pUAST-attB using NotI and Acc65I restriction sites. The construct was injected by Bestgene in an attP line to generate transgenic flies.

RPTP69dExtra:
The N-terminal region of RPTP69d lacking the transmembrane domain (residues 1-796) including a V5 tag was amplified by PCR and cloned into pUAST-attB using the same strategy as above.
Robo3-[fraFN]-GFP:
To generate a Robo3 resistant to Kuzbanian mediated cleavage as it has been described for Robo1 (Coleman et al., 2010), the fibronectin III (FNIII) domains of Robo3 were replaced by the first three Frazzed FNIII. The construct was tagged in the C-Terminal domain with EGFP and cloned in pCDNA3 using StuI restriction sites.

In situ hybridization

In situ hybridization was carried out on L3 brains fixed with 4% formaldehyde using digoxigenin-labeled antisense RNA probes according to standard protocols. To synthesize the probes we amplified a fraction of Slit from genomic DNA using the oligos GTGGGTAAAGTGGTCCATGC and AAGCTGATCCTGTCCACACC. This PCR product was cloned in pGEM-T (promega) then linearize with SpeI and transcribed from T7 promoter for antisense synthesis.

Antibody staining of *Drosophila* brains

For adult and larval brain staining. Animals were dissected in PBS and fixed in PBT 4% formaldehyde for 15 to 20 min. Fixed brains were washed three times for 15 to 20 min in PBT and incubated with the PAXDG buffer (PBT, 5% normal goat serum, 1% BSA, 0.3% deoxycholate) for 30 min to 1 hr at room temperature. Primary antibody incubation was done in PAXDG overnight at 4°C. Then the samples were washed three times with PBT and incubated with the appropriate secondary antibody in PAXDG for 2 to 4 hr, washed with PBT and mounted using the Vectashield mounting medium (Vector, Burlingame, CA, USA). The following antibodies were obtained from the Developmental Studies Hybridoma Bank (DSHB, Iowa city, IA, USA): mouse anti-Slit (1:20), mouse anti-Robo (1:50), mouse anti-Robo3 extracellular (1:50), mouse anti-PDH (1:50), mouse anti-Fasciclin2 (1:50), rat anti N-Cadherin (1:10). Other antibodies used were: rabbit anti-NetA and rabbit anti-Robo2 (gifts from Barry Dickson;1:1000 and 1:500 respectively) mouse anti-GFP 3E6 (Invitrogen, catalog number A11120, 1:250), rabbit anti-GFP (Invitrogen, Carlsbad, CA, USA, catalog number A11122, 1:500) rabbit anti-Robo2 (1:1000), rabbit anti-DsRed (Clontech, Mountain View, CA, USA, catalog number 632496; 1:500) and anti-HRP (Cy5-conjugated Jackson ImmunoResearch 1:50). Secondary antibodies conjugated with Alexa 488, Alexa 555 and Alexa 647 were obtained from Invitrogen and used at 1:500.

Antibody staining of *Drosophila* primary neuron culture

Drosophila primary neuron cultures were generated as described previously (Goncalves-Pimentel et al., 2011; Sanchez-Soriano et al., 2010). In brief, stage 11 embryos (6-7 hrs AEL at 25°C) were homogenized, treated for 5 min at 37°C with dispersion medium, washed and dissolved in Schneider's medium (Schneider, 1964). Then, the aliquots were transferred to coverslips, kept as hanging drop cultures in airtight special culture chambers for 6 hrs at 26°C. For immunocytochemistry, cells were fixed (30' in 4% paraformaldehyde in 0.05 M phosphate buffer, pH 7.2), washed in PBS, and then incubated with antisera diluted in PBS, washed and incubated with the secondary antibody. After several washes in PBS the samples were mounted in vectashield.

Robo3 cell surface biotinylation

To quantify the levels of Robo3 at the surface we performed cell surface biotinylation assay. COS-1 cells were seeded in 10cm dishes and transfected using ViafectTM transfection reagent with 10µ of pCDNA3-Robo3-GFP, or pCDNA3-Robo3-Uncleavable-GFP in presence or absence of pUAST-RPTP69d-V5. After 48h of transfection cells were washed 4 times in PBS pH 8.0 and then subsequently incubated for 30minutes slowly rocking at 4ºC with 0,5 mg/ml Biotine. At the end of the incubation time the cells were washed again in PBS pH 8.0 and quenched for 15 min with 3 ml PBS, 100 mM Glycine, 0.5% BSA fraction V, at 4ºC on slow rocking. After a 3 washes in PBS, 100 mM Glycine the cells were scraped in 1ml of Lysis buffer (50 mM HEPES, pH 7.2, 100 mM NaCl, 1% Triton, + proteases inhibitor), passed through a 26GA3/8 needle and incubated for 20 minutes on ice. The lysates were subsequently spinned at 4ºC for 15 minutes at max speed and the concentration of the supernatant was determined using the Bradford colorimetric assay. 40µg of proteins were kept as input and diluted in Laemly Buffer, while 600µg were incubated with 35 µl of Streptavidine beads Overnight at 4ºC. The following day the beads were

washed 4 times by spinning 1 min, 4000 rpm at 4°C and resuspending in 1ml of lysis buffer. After the last was the beads were spinned at max speed for 1 minute and resuspended in 15µl of 2x Laemly buffer.
This assay was performed also in Hela cells using the same protocol but two 10cm dishes per condition.

Antibody feeding assay
HEK-293T cells were seeded in a 12 well containing Poly-D-lysing coated glass coverslips and transfected using Fugene with 1µg of pCDNA3-Robo3-GFP and pUAST-RPTP69d-V5. After 48h of transfection cells were washed once in PBS and incubated slowly rocking at 4ºC with 500µl of mouse anti-Robo-Extracellular (Hybridoma Bank) diluted 1/100 in PBS. After 1h the cells were washed 3 times in PBS and fixed in 4% PFA for 20 minutes at RT. The PFA was subsequently washed 3 times with PBS and the cells were permeabilized for 10 minutes with 0,1% Triton X-100. Upon 3 washes in PBS the cells were incubated with secondary antibody anti-mouse Alexa 555 and DAPI for 30 minutes at room temperature. Cells were finally mounted on slides with a drop of Glycergel Mounting Medium (Dako) and subsequently imaged on a Nikon confocal.

Imaging
Imaging was performed using a Leica SP5 and SP6 confocal microscopes (Wetzlar, Germany). Images were processed using the ImageJ software (National Institutes of Health, Bethesda, MD, USA). Figures were prepared using Adobe Photoshop (Adobe, San Jose, CA, USA).

Image intensity quantification
Quantification of Slit protein:
Images of adult brains stained with Slit antibody in control and Slit knock down condition were quantified using Image J. For each image, the signal was measure in the calyx and background measure in the ventral region of the brain was subtracted.
Robo3 measurements in cultured neurons:
To quantify the levels of Robo3 at the surface we use anti-Robo3 extracellular, in the absence of detergent. Surface Robo3 was quantified in FIJI (Schindelin et al., 2012), by drawing a line along the outline of axons and measuring the mean intensities within. Each mean value of surface Robo3 was divided by the mean of total overexpressed Robo3. The levels of total overexpressed Robo3 were obtained by measuring the mean intensity of GFP in FIJI as indicated above.
Antibody feeding analysis:
For analysis, the degree of colocalization was determined using Fiji/ImageJ2 macro implemented in Jython. Raw images were imported using BioFormats library. Individual cells were manually segmented in each image slice by the user. Stack threshold levels for each channel were calculated using preselected autothresholding algorithms available in Fiji (Yan for the green channel and Max Entropy for the red channel). Determined threshold levels were used to calculate Mander's overlap coefficient using ImgLib2 implementation of the colocalization algorithm. Code for the macro is available on GitHub (https://github.com/rejsmont/FijiScripts/blob/master/mColoc3D.py).

Immunoprecipitation
S2 cells were cultured in 6 well plates at 25C using in Sf900II medium. Electroporation was carried out using the Amaxa V kit (Lonza, Basel, Switzerland). The constructs used were pMT-Gal4, pUAST-GFP, pUAST-Robo3-GFP (Yasushi Hiromi), and pUAST-RPTP69d-V5. For Slit treatment, Slit was obtained from the supernatant of S2 stable-expressing cells and was added 3 days after transfection. 6 hours later cells were extracted and the pellet frozen.
Cells were lysed using 400 ul of Ripa buffer (50mM Tris pH: 7,5; Glycerol 10%; NP-40 0,4%; 150mM NaCl and fresh added protease inhibitors cocktail 100x (Amresco). NaF and Na3VO4 phosphatase inhibitors were added if detection of tyrosine phosphorylation was required) per well (from a 6 well plate). 25 ul of anti-GFP conjugated beads (Chromotek) were washed 3 times with Ripa buffer. Then, 250 ul of cell lysate was mixed the beads and the volume brought to 1 ml, and then incubated 2hrs at 4C in a rotator, followed by 4 washes with Ripa buffer 10 min each. Finally, the elution was performed with 15 ul of laemmeli Buffer (2x) followed by boiling 5 min at 95C and stored at -20C.

Western Blotting
SDS-PAGE was performed using 4-12% gradient polyacrylamide gels and then transferred to nitrocellulose membranes. For blotting, Mouse anti-V5 cat no R96025 (1:500, Invitrogen-Life Technologies) , Mouse anti-Phospho-Tyrosine clone 4G10 cat no 05-321 (Millipore), Rabbit anti-GFP cat no A6455 (1:1000, Invitrogen-Life

Technologies) , Slit antibody (1:100, DSHB, Iowa city, IA, USA) and Mouse anti-actin cat no ab3280 (Abcam) were used. Secondary antibodies were anti-rabbit HRP or anti-mouse-HRP (Jackson). Visualization of the samples was performed using ECL Western Blotting Detection System (GE Healthcare, UK). Quantifications Robo3 processing were performed by densitometry of the blots. Total Robo was determined by addition of the full length and processed form normalized by the actin loading control. Then the ratio full length v/s total was plotted.

Statistics

Statistical analysis was performed using the Prism software (GraphPad Software Inc, La Jolla, CA, USA). For sLNv measurements, results are presented as arbitrary units (A.U.) representing the fraction between the lengths of the dorsal projections divided by the distance between cell bodies in the two brain hemispheres. For analysis a two-tailed t-test was used for two-group comparisons and ANOVA for multiple comparisons. For primary neuronal culture experiments the Mann-Whitney test was used. For analysis of Robo phenotypes in DCN neurons a Fisher Exact test was performed.

Supplemental References

Desai, C.J., Gindhart, J.G., Jr., Goldstein, L.S., and Zinn, K. (1996). Receptor tyrosine phosphatases are required for motor axon guidance in the Drosophila embryo. Cell *84*, 599-609.

Goncalves-Pimentel, C., Gombos, R., Mihaly, J., Sanchez-Soriano, N., and Prokop, A. (2011). Dissecting regulatory networks of filopodia formation in a Drosophila growth cone model. PloS one *6*, e18340.

Sanchez-Soriano, N., Goncalves-Pimentel, C., Beaven, R., Haessler, U., Ofner-Ziegenfuss, L., Ballestrem, C., and Prokop, A. (2010). Drosophila growth cones: a genetically tractable platform for the analysis of axonal growth dynamics. Developmental neurobiology *70*, 58-71.

Schindelin, J., Arganda-Carreras, I., Frise, E., Kaynig, V., Longair, M., Pietzsch, T., Preibisch, S., Rueden, C., Saalfeld, S., Schmid, B., *et al.* (2012). Fiji: an open-source platform for biological-image analysis. Nat Methods *9*, 676-682.

Schneider, I. (1964). Differentiation of Larval Drosophila Eye-Antennal Discs in Vitro. The Journal of experimental zoology *156*, 91-103.
